# Development and Evaluation of a Novel Inhalable Liposomal Powder Co-Encapsulating ASSNAC and Pirfenidone via Spray Freeze-Drying for Targeted Pulmonary Fibrosis Therapy

**DOI:** 10.3390/ph19091374

**Published:** 2026-08-31

**Authors:** Qinxiu Zhang, Shouwei Sun, Miaomiao Lu, Runxin Qin, Junxuan Ren, Lianjie Yao, Dianlong Jia, Jinjie Chang, Xiaohong Chu, Rui Wang, Fang Liu, Jun Li

**Affiliations:** 1 School of Pharmaceutical Sciences and Food Engineering, Liaocheng University, Hunan Road, Liaocheng 252000, China; zhangqinxiu@lcu.edu.cn (Q.Z.);; 2 Shandong Provincial Key Laboratory of Applied Technology for Protein and Peptide Drugs, Liaocheng 252000, China; 3 State Key Laboratory of Macromolecular Drugs and Large-Scale Preparation, School of Pharmaceutical Sciences and Food Engineering, Liaocheng 252000, China

**Keywords:** pulmonary fibrosis, pirfenidone, ASSNAC, spray freeze-drying, dry powder inhaler

## Abstract

**Background:** Pulmonary fibrosis (PF) is a progressive lung disease with limited therapies. Oral Pirfenidone (PFD), an approved anti-fibrotic, shows poor lung bioavailability and significant toxicity. S-Allylmercapto-N-acetylcysteine (ASSNAC) has been demonstrated to possess significant anti-inflammatory and antioxidant properties. **Methods:** In this study, dry powder inhaler formulations (DPIs) co-encapsulating ASSNAC and PFD in liposomes are reported. The formulation was prepared via spray freeze-drying (SFD) using L-leucine as both a cryoprotectant and a surface morphology modifier. The interfacial enrichment of L-leucine during atomization and freezing created a hydrophobic, corrugated surface that lowered particle surface energy, prevented liquid/solid bridge formation, and reduced hygroscopicity. **Results:** The resulting liposomal powders exhibited optimal aerosol performance: fine particle fraction (FPF) of 61.08% ± 2.45% and mass median aerodynamic diameter (MMAD) of 2.22 ± 0.11 µm. Cellular studies demonstrated that dual-loaded liposomes were efficiently taken up by Beas-2B and HFL-1 cells, with no cytotoxicity (cell viability > 90%) and negligible hemolysis. In the TGF-β1-induced in vitro fibrosis model, the combined treatment exerted prominent therapeutic effects. This regimen preserved normal epithelial morphology, accelerated wound repair, suppressed fibroblast invasiveness, and lowered the expression levels of Collagen I, Collagen III, and α-SMA. In bleomycin-induced pulmonary fibrosis rats, inhaled ASSNAC + PFD liposomal powder markedly relieved lung tissue injury and collagen accumulation. It restored redox balance and lowered pulmonary TGF-β1, hydroxyproline and collagen III levels. The formulation blocked TGF-β1 signaling and fibrotic gene expression. **Conclusions:** Pulmonary administration avoids oral pirfenidone-induced liver and stomach toxicity. Collectively, the ASSNAC + PFD co-encapsulated liposomal dry powder produced by SFD represents a safe and efficacious therapeutic strategy for PF.

## 1. Introduction

Pulmonary fibrosis (PF) is a chronic progressive lung disease. It is characterized by imbalanced epithelial–mesenchymal transition, massive extracellular matrix accumulation, abnormal immune cell recruitment, and irreversible lung scarring, ultimately leading to loss of lung function and even death [1,2,3,4]. Cough, sputum production, and dyspnea are the main early symptoms of pulmonary fibrosis [5]. As the disease worsens, it progresses to the stage of extensive pulmonary interstitial fibrosis, which has a severe impact on the respiratory function of the patients and eventually leads to respiratory failure and death [5]. The main onset age of PF is 50–70 years, with high mortality and median survival of only 2–5 years [6]. PF is also a characteristic of lung injury in severe COVID-19 patients [7].

Pulmonary fibrosis develops in three phases. Lung epithelial injury disrupts redox balance, triggering macrophage recruitment and inflammatory factor release. This drives fibroblast differentiation, proliferation and collagen buildup, causing excessive ECM deposition and impaired lung function [8,9]. Therefore, relevant targets such as antioxidant, anti-inflammatory and anti-fibrotic therapies can be offered as treatment strategies for pulmonary fibrosis according to different stages [10,11].

Pirfenidone (PFD) capsules and nintedanib (NDNB) soft capsules are the current drugs approved by the FDA for the treatment of pulmonary fibrosis, which slow the progression of pulmonary fibrosis [12,13]. Although PFD is generally well-tolerated, clinical studies have shown that a minority of patients discontinue therapy due to various adverse effects following oral administration, including headache, dyspepsia, nausea, diarrhea, vomiting, fever, hepatic dysfunction, dizziness, skin-related adverse effects, and facial paralysis [14,15,16]. Therefore, inhalation or intrapulmonary delivery of PFD serves as a promising alternative to optimize administration routes, lower therapeutic dosage, alleviate adverse reactions, and reduce off-target systemic effects [17,18].

PFD can regulate the activity of transforming growth factor-β (TGF-β) and tumor necrosis factor-α (TNF-α), leading to the inhibition of fibroblast proliferation and collagen synthesis [19]. In antioxidant effects, it has been shown that PFD could scavenge ROS by inhibiting nicotinamide adenine dinucleotide phosphate (NADPH)-dependent lipid peroxidation [20,21]. S-Allylmercapto-N-acetylcysteine (ASSNAC) is an organosulfur compound and a derivative of N-Acetylcysteine (NAC). Studies have shown that ASSNAC has anti-inflammatory and antioxidative effects and has been used in the treatment of chronic obstructive pulmonary disease and pulmonary fibrosis [22,23]. Studies have shown that ASSNAC generates anti-fibrotic effects through anti-inflammatory and antioxidant activities [24]. It mainly generated antioxidant effects by providing GSH to the body and activating the Nrf2/HO-1 signaling pathway [24]. ASSNAC, the special organosulfur compound, is able to inhibit the expression of Toll-like receptor 4 (TLR-4), which further blocks the NF-κB signaling pathway and thereby mediates anti-inflammatory effects [25]. Therefore, the combination of ASSNAC and PFD is pharmacologically rational, offering a multi-targeted therapeutic approach by simultaneously enhancing antioxidant defense and suppressing inflammatory and fibrotic pathways.

Despite the therapeutic potential of combination therapy, effective delivery of both ASSNAC and PFD to the deep lungs remains a significant challenge. The pulmonary microenvironment in patients with pulmonary fibrosis is notably complex, characterized by mucus barriers, phagocytic clearance by macrophages, biological barriers, and potential irritation from inhaled agents [26,27]. Liposomes composed of phospholipids, which are analogous to cell membranes and lung surfactants, can enhance drug biocompatibility and mitigate pulmonary irritation [28,29]. Additionally, DSPE-PEG 2000 modification improves mucus permeability and reduces macrophage-mediated phagocytic clearance [27,30]. Therefore, liposomal encapsulation represents a promising strategy to improve the bioavailability and therapeutic efficacy of the combined drugs.

The potential of liposomal encapsulation as a targeted delivery strategy is constrained by the poor stability of aqueous dispersions, a critical barrier to their long-term storage and clinical translation [31]. These systems are prone to several destabilizing phenomena during storage, including phospholipid oxidation, hydrolysis, particle aggregation, fusion, and drug leakage [32,33]. These detrimental processes ultimately curtail shelf life and jeopardize both therapeutic efficacy and patient safety [34].

Freeze-drying is widely used to improve liposome stability, but conventional freeze-drying is time-consuming and can damage lipid bilayers via ice crystal formation during slow freezing. Spray freeze-drying (SFD) addresses these issues: it rapidly atomizes liposomal suspension into a cryogenic medium for instantaneous droplet freezing, minimizing destructive ice crystal growth and better preserving liposome structural integrity and lamellarity.

Lyoprotectants are necessary to maintain phospholipid-bilayer integrity and avoid membrane fusion in lyophilization. Mostly carbohydrates, polyols and amino acids, they preserve native lipid structure. Even with protectants, freeze-dried liposome powder still has drawbacks like poor morphology, weak dispersibility, particle aggregation and drug leakage [35,36]. L-leucine, a naturally occurring hydrophobic amino acid, has garnered significant attention as a multifunctional excipient in dry powder inhaler formulations (DPIs) [37,38]. Its hydrophobic side chains interact favorably with the phospholipid bilayer, preventing fusion and aggregation upon rehydration. From an aerosol performance perspective, L-leucine modifies the surface topology of the particles [39,40]. Upon sublimation of the ice matrix during drying, L-leucine crystallizes to form a porous, “raspberry-like” or corrugated surface morphology [40]. This specific morphology reduces particle density and interparticulate cohesive forces, significantly enhancing the flowability and dispersibility of the powder [41]. Consequently, this facilitates deep lung deposition upon inhalation.

Therefore, this study aimed to develop a novel co-encapsulated liposomal system for ASSNAC and PFD, which is then converted via SFD into an inhalable powder formulation for PF. The dual-drug strategy targets oxidative stress, inflammation, and fibrosis. The liposomes were prepared by the SFD process with L-leucine as a cryoprotectant and morphology modifier. This approach ensures liposomal stability and generates particles with optimal aerodynamic properties. The dry powder inhaler formulation provides a non-invasive delivery route to maximize therapeutic efficacy while minimizing systemic toxicity.

## 2. Results and Discussion

### 2.1. Characterization of ASSNAC + PFD/Lip and DPIs

#### 2.1.1. Characterization of ASSNAC + PFD/Lip

Co-loaded liposomes were prepared by the calcium acetate method. In the co-loaded liposomes, the EE for ASSNAC and PFD were 95.23% ± 2.35% and 70.20% ± 2.90%, and the DL for ASSNAC and PFD were 9.52% ± 0.13% and 5.50% ± 0.39%, respectively (Supplementary Information Table S1).

**Figure 1:**
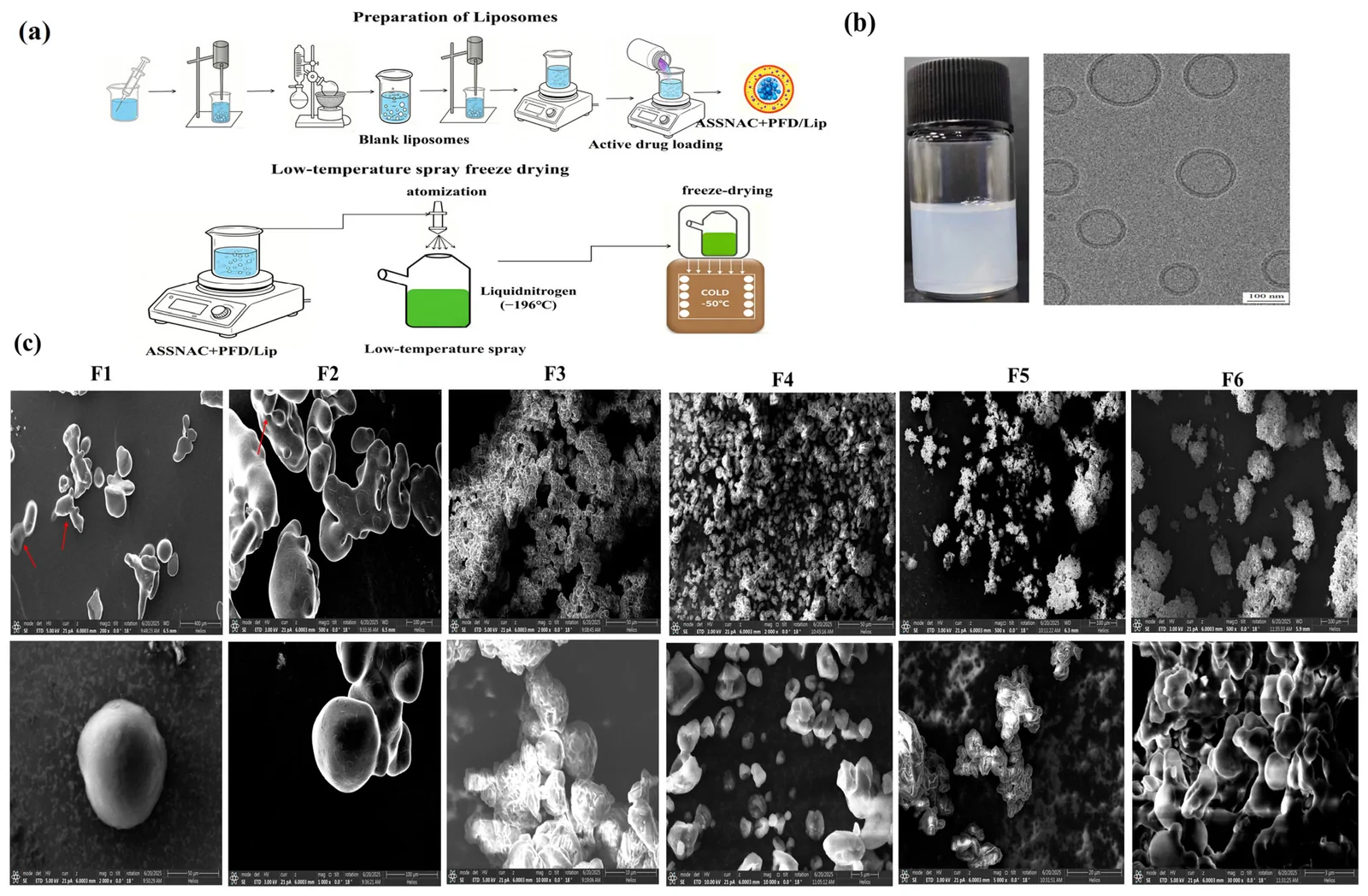
Preparation and characterization of the ASSNAC + PFD liposomes and DPIs. (**a**) Preparation process of ASSNAC + PFD/Lip and ASSNAC + PFD/DPIs. (**b**) Macroscopic appearance of ASSNAC + PFD/Lip and TEM image of the ASSNAC + PFD/Lip. (**c**) SEM images of DPIs prepared with different formulations. Upper-row micrographs: magnification ×1000, scale bar, 100 μm; bottom-row micrographs: magnification ×10,000, scale bar, 10 μm.

**Figure 2:**
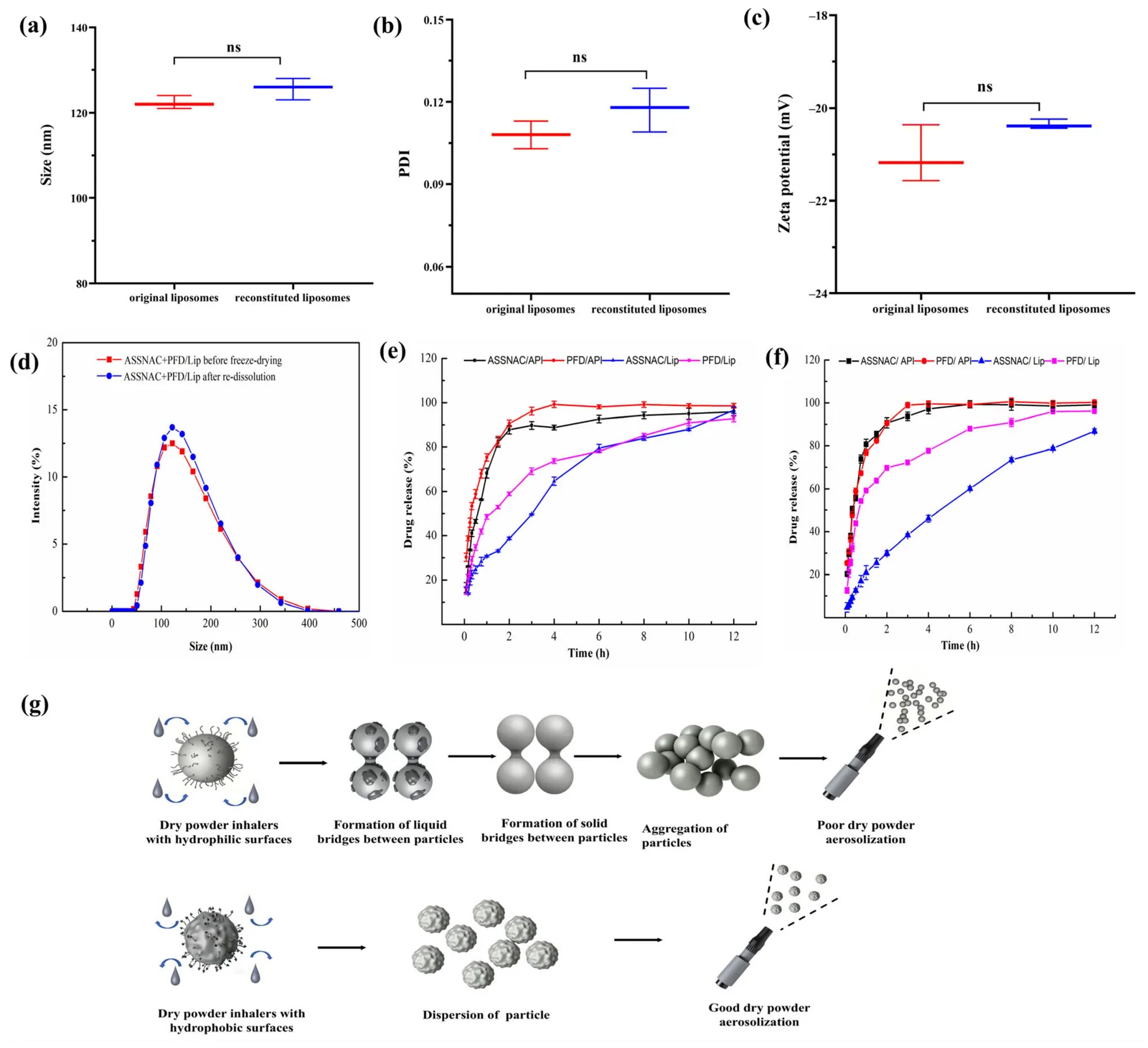
Comprehensive physicochemical characterization of ASSNAC + PFD liposomes and DPIs. (**a**–**d**) Particle size, PDI, zeta potential and particle size distribution of ASSNAC + PFD/Lip before freeze-drying and after redispersion. (**e**) Drug release at pH 5.5. (**f**) Drug release at pH 7.4. (**g**) Schematic diagram of the effect of powder surface hydrophilicity/hydrophobicity on the aerosolization performance of DPIs. ns: not significant.

As shown in Figure 1a, blank liposomes were prepared using the calcium acetate gradient method, followed by active loading of ASSNAC and PFD to obtain ASSNAC + PFD/Lip. The ASSNAC + PFD/Lip was then converted into an inhalable dry powder via spray freeze-drying. As shown in Figure 1b, the liposomal dispersion appeared as a translucent, bluish opalescent solution, indicating a colloidal system with particle sizes smaller than the wavelength of visible light. Cryo-TEM images revealed that the liposomes possessed a typical spherical vesicle structure with a uniform size distribution. The particle size, PDI, zeta potential and particle size distribution of ASSNAC + PFD/Lip before freeze-drying and after re-dissolution of DPIs are shown in Figure 2a–d (Supplementary Table S3). The particle size profiles of the two formulations remained similar before and after lyophilization, with a mean diameter of approximately 120 nm. The size, PDI, and zeta potential of re-dissolved liposomes almost matched those of liposomes before freeze-drying, indicating that the SFD process did not induce significant aggregation, fusion, or rupture of the liposomes. The preserved particle size after lyophilization further confirmed the protective effect of SFD, which minimizes ice crystal growth and maintains the integrity of the lipid bilayer. This also demonstrated that L-leucine effectively prevented interparticulate fusion during drying. Such stable size is essential for consistent aerosol performance and deep lung deposition.

#### 2.1.2. Effect of L-Leucine on Particle Morphology and Dispersibility

The morphology of DPIs was examined by SEM. As illustrated in Figure 2g, conventional dry powder systems lacking appropriate excipients typically exhibited hydrophilic particle surfaces, which promoted the formation of liquid bridges between particles during drying. The subsequent solidification of these liquid bridges led to irreversible particle aggregation and poor aerosolization performance. To overcome this issue, L-leucine was introduced as a surface-active excipient. The excipient composition of DPIs was presented in Table 1. Figure 1c presents SEM images of six formulations (F1–F6). Formulations without L-leucine (F1–F3) exhibited severe aggregation into irregular fused masses, attributable to the hygroscopicity of the saccharide matrix and unmitigated interparticle cohesion. In contrast, formulations supplemented with 10% L-leucine (F4–F6) displayed well-dispersed, spherical particles with rough, wrinkled surfaces. Among these, F6 (containing both mannitol and L-leucine) showed optimal morphology, characterized by the highest degree of sphericity, uniform size distribution, and minimal aggregation.

The superior performance of L-leucine can be attributed to its distinctive physicochemical behavior during SFD. Driven by its hydrophobic isobutyl side chain, L-leucine rapidly migrates to the air–liquid interface of atomized droplets. During instantaneous freezing in liquid nitrogen, it is excluded from growing ice crystals and accumulates at the droplet surface. Upon sublimation, L-leucine crystallizes into a nanoscale, corrugated “raspberry-like” morphology (Figure 1c, F6). This unique structure confers multiple advantages: reduced interparticle contact area, lower surface energy, and a hydrophobic barrier. Furthermore, the hydrophobic side chains effectively prevent the formation of liquid/solid bridges (Figure 2g), the primary cause of irreversible aggregation under high humidity. Consequently, L-leucine preserves particle dispersibility during drying and storage, and facilitates deep lung deposition. Collectively, these results demonstrate that L-leucine is an ideal excipient for inhalable dry powder formulations.

#### 2.1.3. Study on Moisture Sorption and Aerodynamic Performance

**Figure 3:**
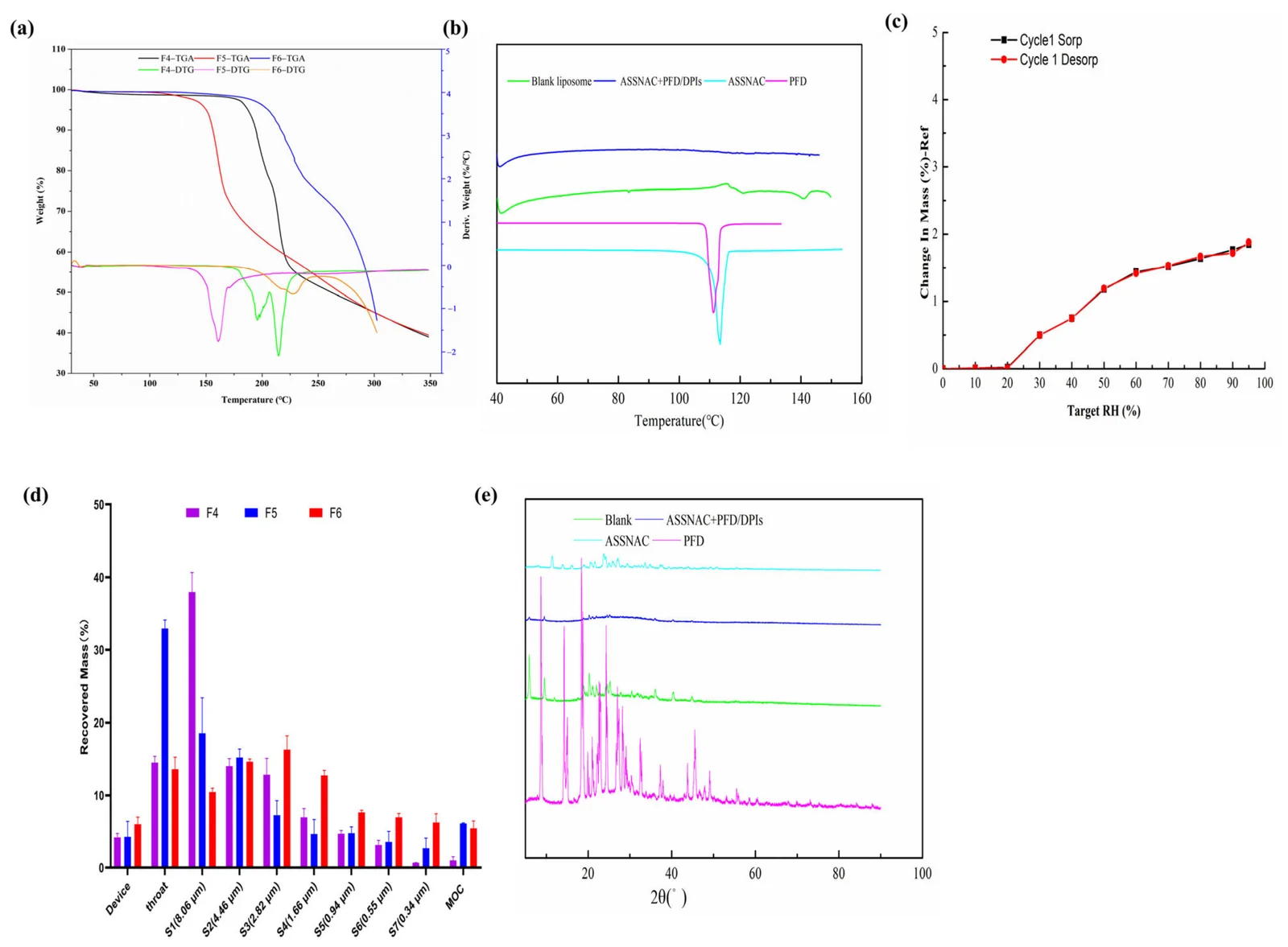
Comprehensive physicochemical characterization of ASSNAC + PFD DPIs. (**a**–**d**). (**a**) TGA and DTG analysis of samples. (**b**) DSC thermograms of samples. (**c**) DVS plot showing the moisture sorption isotherm of the optimized powder at varying relative humidity. (**d**) Aerodynamic particle size distribution and deposition profile of the powder in an NGI for F4, F5, and F6. (**e**) XRD results of samples (*n* = 3 independent experiments).

The stability of DPIs is heavily influenced by humidity. As schematically illustrated in Figure 2g, hydrophilic particle surfaces promote the formation of liquid bridges under humid conditions, leading to irreversible aggregation. To assess the moisture resistance conferred by L-leucine, DVS analysis was performed on the optimal formulation F6. The DVS isotherm showed that F6 exhibited low hygroscopicity below 90% RH, with a mass increase of less than 5% (Figure 3c). This behavior is largely attributed to the hydrophobic nature of L-leucine enriched on the particle surface, which acts as a physical moisture barrier and prevents the formation of liquid/solid bridges [42]. Collectively, the DVS results corroborate the morphological observations (Figure 1c) and confirm that L-leucine effectively mitigates humidity-driven aggregation, thereby preserving powder dispersibility and aerosol performance.

The aerodynamic particle size distribution and deposition behavior of formulations F4, F5, and F6 were evaluated using NGI. The stage-wise mass deposition was shown in Figure 3d, and the corresponding aerodynamic parameters (MMAD, GSD, FPF, ED) were summarized in Supplementary Table S2. All three formulations exhibited low mass retention in the throat and device (emitted dose > 94%), indicating efficient powder deagglomeration and release from the inhaler device. Among the tested formulations, F6 exhibited the most favorable aerodynamic properties, with an MMAD of 2.22 ± 0.11 µm and a GSD of 1.98 ± 0.07, supporting its potential for deep lung delivery. The fine particle fraction (FPF, < 5 µm) of F6 was 61.08 ± 2.45%, significantly higher than those of F4 (31.91%) and F5 (24.97%). Consistently, Figure 3d showed that the majority of F6 particles accumulated in stages 2–5, which correspond to aerodynamic diameters below 5 µm. This superior aerosol performance was primarily attributed to the optimal particle morphology of F6 (Figure 1c), characterized by a corrugated, wrinkled surface due to the combined effect of mannitol and L-leucine, which reduced interparticle cohesion and enhanced dispersibility. These results confirmed that the optimized F6 dry powder formulation is capable of bypassing upper respiratory tract deposition and delivering ASSNAC + PFD/Lip effectively to the alveolar region, the primary target site for pulmonary fibrosis therapy.

#### 2.1.4. In Vitro Release Studies

In view of the weakly acidic microenvironment in pulmonary fibrosis, and to simulate the conditions following inhalation, release profiles were measured at both pH 5.5 and pH 7.4 [43,44]. As shown in Figure 2e–f, the cumulative release patterns of all formulations at pH 5.5 were generally comparable to those observed at pH 7.4. In both media, free APIs exhibited a relatively rapid release, whereas liposomal formulations displayed a sustained and continuous release over the entire observation period. To elucidate the release mechanism, the cumulative data were fitted to zero-order, first-order, Higuchi, Korsmeyer–Peppas and Weibull models. Free APIs relied on quick dissolution-dominated release. The bilayer-embedded ASSNAC/Lip followed non-Fickian anomalous diffusion controlled by phospholipid-bilayer swelling with barely observable burst leakage. The aqueous-core-encapsulated PFD/Lip escaped from liposomes via standard Fickian transmembrane diffusion. These results demonstrate that liposomal encapsulation transforms the fast dissolution of raw drugs into matrix-mediated sustained release. Varied drug-loading sites inside vesicles produce divergent diffusion behaviors. Detailed fitting parameters were presented in Supplementary Tables S4 and S5.

#### 2.1.5. Physical State of Encapsulated Drugs Revealed by TGA/DTG, DSC and XRD

To investigate the physical state of the drugs within the matrix, TGA, DSC and XRD were performed. TGA (Figure 3a) was performed to assess residual moisture and thermal stability of F4, F5, and F6. Benefiting from the incorporation of L-leucine, all three formulations exhibited extremely low moisture content. Negligible weight loss was observed below 120 °C, confirming favorable thermal stability under conventional storage conditions. The main decomposition peaks were observed at 195.5 °C and 214.7 °C for F4, 160.1 °C for F5, 209.7 °C and 227.2 °C for F6. Therefore, the results indicate that all three formulations possess adequate thermal robustness for processing and storage. The Δm and T_max_ are presented in Table 1.

The DSC thermograms (Figure 3b) corroborated these findings; the endothermic melting peak of crystalline PFD and ASSNAC was absent in the liposomal formulations, further confirming the successful encapsulation and amorphous state of the drug. As shown in the XRD patterns (Figure 3e), pure PFD exhibited sharp, intense diffraction peaks, indicating a crystalline nature. However, these characteristic peaks disappeared in the ASSNAC + PFD/Lip and blank liposome samples, suggesting that the drugs were molecularly dispersed or in an amorphous state within the liposomal bilayer and the amorphous excipient matrix. This amorphization was beneficial for drug release and bioavailability.

### 2.2. Cellular Uptake, Hemocompatibility, and Cytotoxicity Evaluation In Vitro

#### 2.2.1. Efficient Cellular Uptake in Pulmonary Cells

**Figure 4:**
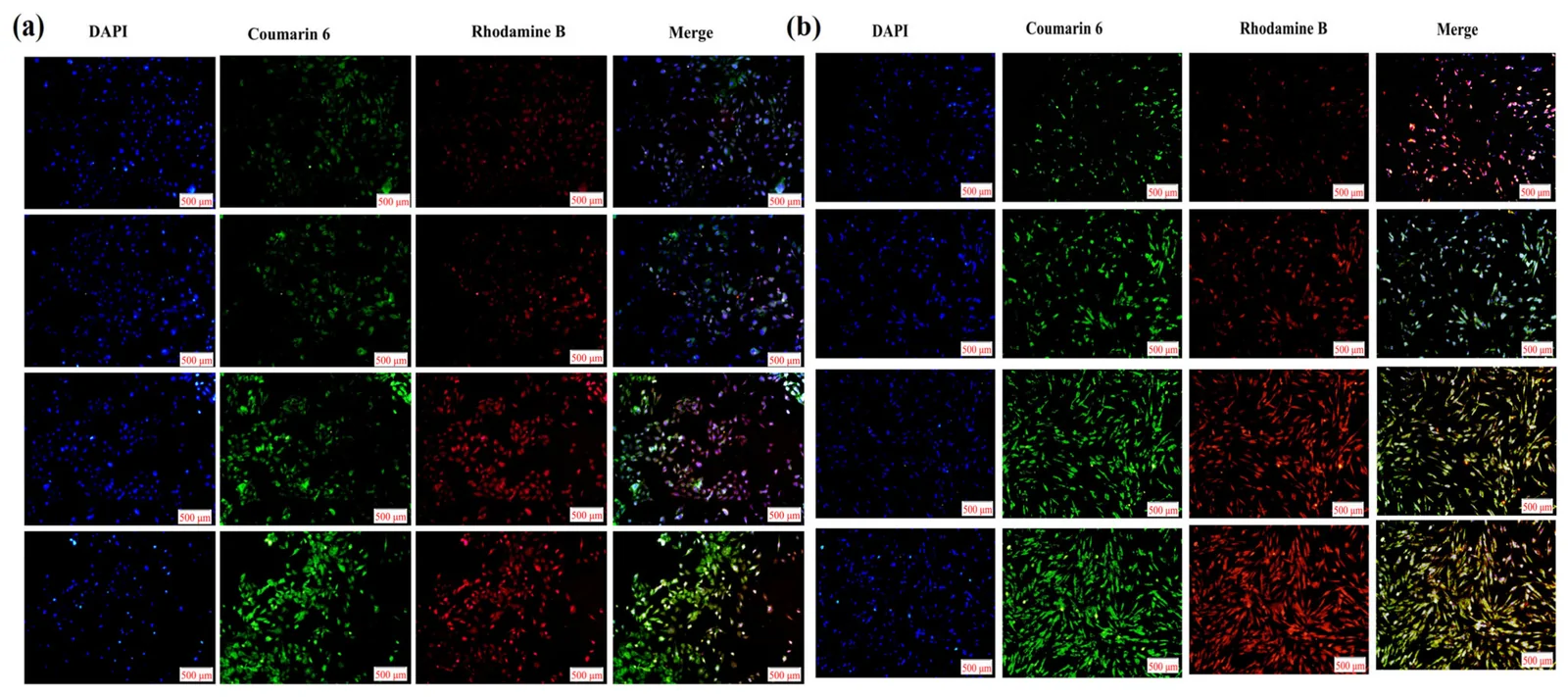
Cellular uptake in vitro. Intracellular uptake of coumarin 6 and rhodamine B loaded liposomes by Beas-2B cells (**a**) and HFL-1 cells (**b**) after incubation for 15, 30, 60 and 120 min (scale bars: 500 μm); (*n* = 3 independent experiments).

To investigate the cellular uptake capacity of DPIs, the uptake of coumarin 6 and rhodamine B-loaded DPIs by HFL-1 and Beas-2B cells was examined. As shown in Figure 4a,b, the uptake of coumarin 6 and rhodamine B liposomes increased significantly over 120 min. These results demonstrated that liposomes were readily taken up by both cell lines, likely due to the similarity between the liposomal phospholipids and the cell membrane.

#### 2.2.2. Hemocompatibility Assessment

**Figure 5:**
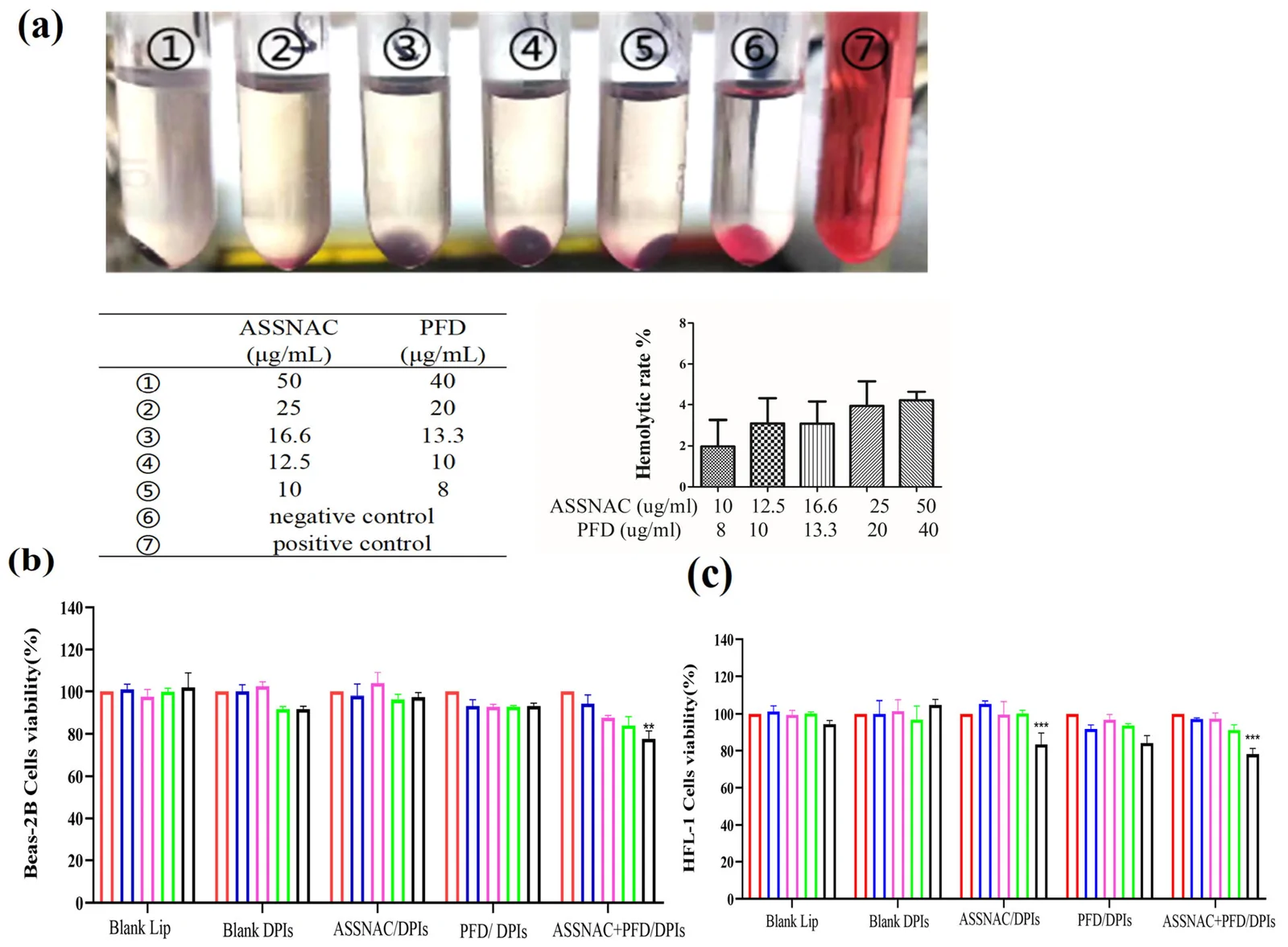
Hemocompatibility and biocompatibility of formulations in vitro. (**a**) Hemolysis assay: Photographs of red blood cells treated with various concentrations of ASSNAC and PFD DPIs, and the corresponding quantitative hemolysis rates; cell viability of Beas-2B cells (**b**) and HFL-1 cells (**c**) treated with different samples. Group settings: blank liposome, blank DPIs, ASSNAC/DPIs (0, 30, 60, 120, and 240 μM), PFD/DPIs (0, 50, 100, 200, and 400 μM), ASSNAC + PFD/DPIs (ASSNAC: 0, 30, 60, 120, and 240 μM; PFD: 0, 50, 100, 200, and 400 μM). Data are presented as the mean ± SD (*n* = 3 independent experiments), ** *p* < 0.01, *** *p* < 0.001 vs. the blank control group.

Given the intended pulmonary administration, hemolysis assays were conducted to ensure the safety of the formulation upon potential contact with blood components. As illustrated in Figure 5a, the supernatants of red blood cells treated with various concentrations of ASSNAC + PFD/DPIs remained clear and colorless, visually indistinguishable from the negative control. In contrast, the positive control showed a bright red color due to complete hemolysis. Quantitative analysis revealed that the hemolysis rates for all tested groups were well below 5%.

#### 2.2.3. In Vitro Cytotoxicity

The cytocompatibility of the blank and drug-loaded formulations was further assessed using standard MTT assays on Beas-2B (Figure 5b) and HFL-1 (Figure 5c) cells. After incubation for 24 h, neither the blank DPIs nor the drug-loaded formulations (ASSNAC, PFD, and their combination) caused a significant reduction in cell viability compared to the untreated control group. Cell viability remained above 90% across all tested concentrations, demonstrating that the excipients and the encapsulated drugs were non-toxic to normal lung cells. This confirmed the biocompatibility of the developed DPI system for pulmonary delivery applications.

### 2.3. Anti-Fibrotic Efficacy Evaluation In Vitro

#### 2.3.1. Protection Against TGF-β1-Induced Epithelial Injury

**Figure 6:**
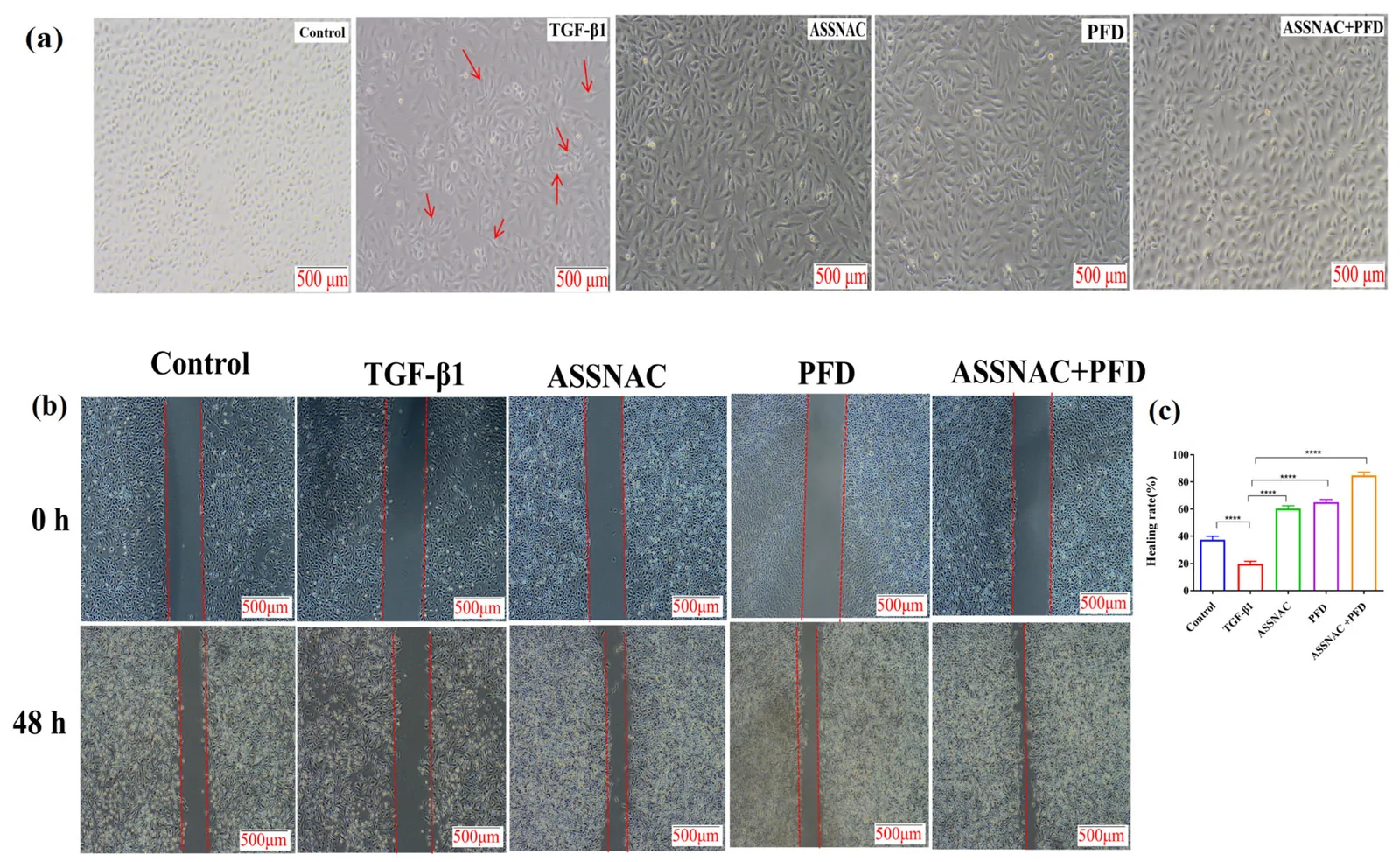
Protective effects of the ASSNAC + PFD/DPIs against TGF-β1-induced epithelial injury and fibroblast activation. (**a**) Representative bright-field microscopy images showing morphological changes in Beas-2B cells following different treatments (control, TGF-β1, ASSNAC, PFD, ASSNAC + PFD). Red arrows indicate spindle-shaped, mesenchymal-like cells induced by TGF-β1 stimulation (scale bars: 500 μm). (**b**) Wound healing assay evaluating the migratory capacity of Beas-2B cells under TGF-β1 stimulation and drug treatments. Micrographs were captured at 0 h and 48 h post-scratching (scale bars: 500 μm). (**c**) Quantitative analysis of the wound healing rate in Beas-2B cells after 48 h of treatment. All data are presented as mean ± SD (*n* = 3 independent experiments). **** *p* < 0.0001.

The maintenance of alveolar epithelial integrity is essential for preventing the initiation of pulmonary fibrosis. Figure 6a depicts the morphological changes in Beas-2B cells under fibrotic conditions. Control cells displayed a characteristic cobblestone-like epithelial morphology. Upon stimulation with TGF-β1, the cells underwent significant morphological deterioration, becoming enlarged and spindle-shaped with retracted intercellular contacts, indicative of epithelial injury and early mesenchymal transition. However, treatment with ASSNAC and PFD effectively preserved the epithelial morphology, preventing the TGF-β1-induced structural disruption. This suggested that the ASSNAC and PFD formulation protects epithelial cells from fibrotic insults, thereby maintaining the structural integrity of the alveolar barrier.

#### 2.3.2. Promotion of Epithelial Cell Migration and Repair

Impaired re-epithelialization is a critical factor in the pathogenesis of pulmonary fibrosis [45]. To evaluate the effect of the formulation on epithelial repair, a wound healing assay was performed using Beas-2B cells (Figure 6b). At 0 h, the scratch widths were consistent across all groups. After 48 h, the TGF-β1 group exhibited significantly delayed wound closure compared to the control, indicating that the fibrotic microenvironment suppresses the migratory capacity of bronchial epithelial cells. Conversely, treatment with ASSNAC markedly accelerated scratch closure. Notably, the ASSNAC + PFD group demonstrated the most rapid migration, with the wound area almost completely closed. These results indicated that the ASSNAC formulation not only targets fibroblasts but also actively promotes the migration and regenerative potential of bronchial epithelial cells, which is essential for restoring the integrity of the alveolar epithelium.

#### 2.3.3. Suppression of Fibroblast Invasion

**Figure 7:**
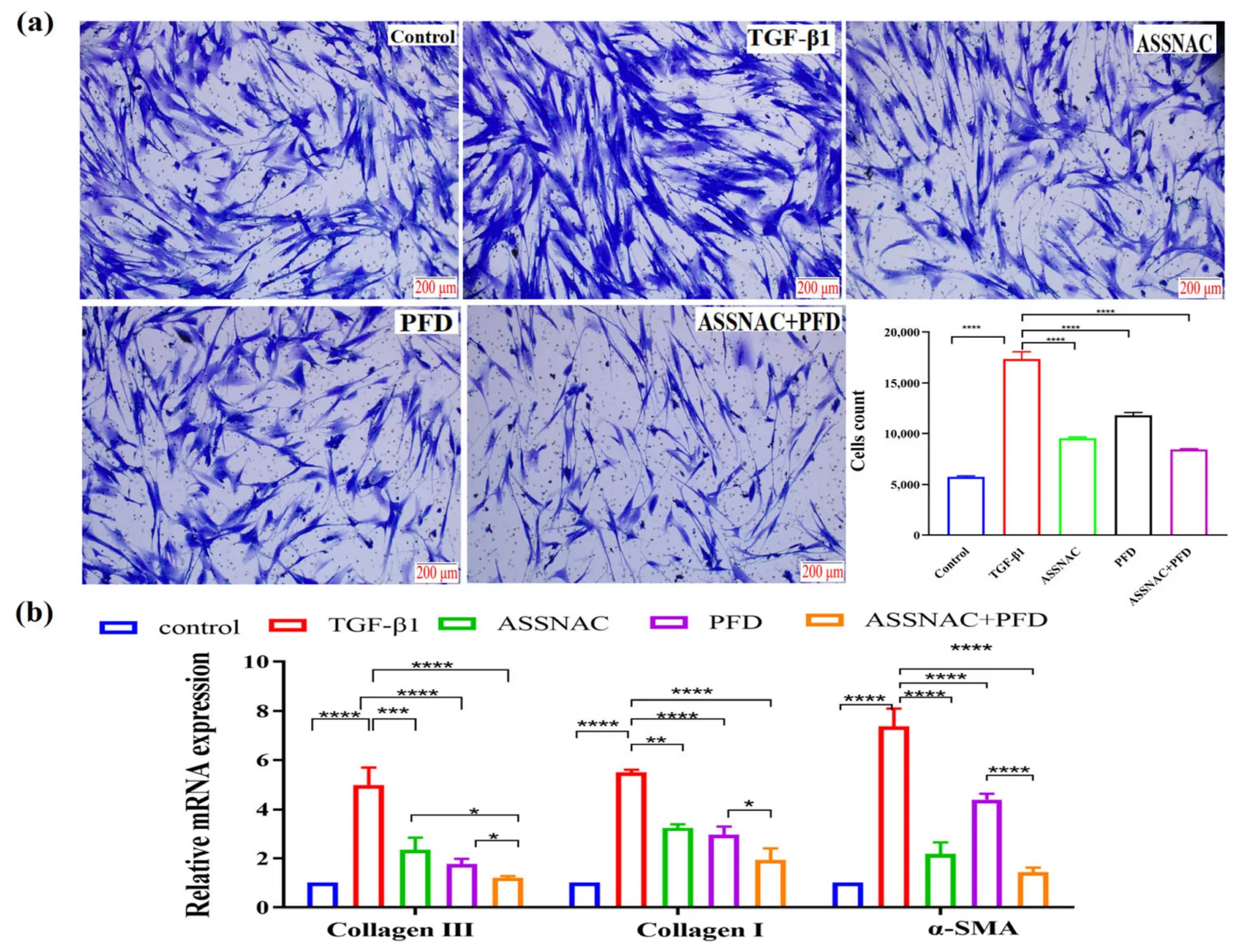
(**a**) Transwell invasion assay assessing the invasive potential of HFL-1 fibroblasts following TGF-β1 stimulation and drug interventions and quantitative analysis (scale bars: 200 μm). (**b**) Relative mRNA expression levels of Collagen III, Collagen I, and α-SMA in HFL-1 cells determined by qRT-PCR. All data are presented as mean ± SD (*n* = 3 independent experiments). * *p* < 0.05, ** *p* < 0.01, *** *p* < 0.001, **** *p* < 0.0001.

The invasive capacity of fibroblasts contributes to the thickening of the alveolar interstitium [46]. As shown in the Transwell invasion assays (Figure 7a), TGF-β1 stimulation resulted in a substantial increase in the number of invasive HFL-1 cells compared to the control group. Both ASSNAC and PFD treatments reduced the number of cells penetrating the membrane. However, the ASSNAC + PFD combination exhibited the most potent inhibitory effect. Quantitative analysis confirmed that the combination therapy significantly suppressed fibroblast invasion more effectively than monotherapies. Collectively, these findings demonstrate that ASSNAC exerts a dual therapeutic mechanism: inhibiting the activation and invasion of fibroblasts while simultaneously enhancing the reparative migration of epithelial cells.

#### 2.3.4. Inhibition of Extracellular Matrix Deposition and Fibrotic Marker Expression

**Figure 8:**
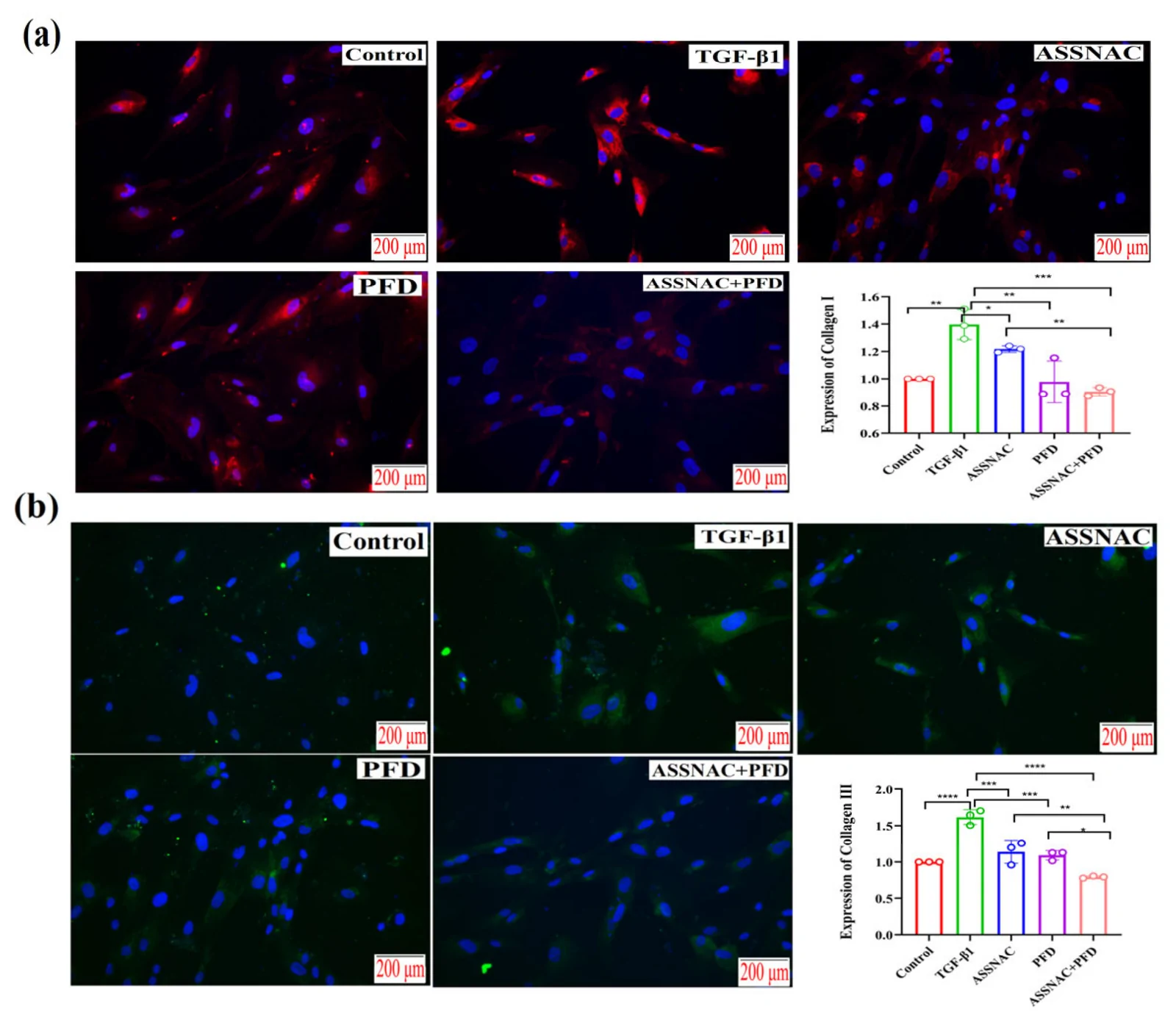
Effects of ASSNAC + PFD/DPIs on TGF-β1-induced collagen and α-SMA expression. (**a**,**b**) The immunofluorescence images of Collagen I (red) and Collagen III (green) in HFL-1 cells treated with TGF-β1 alone or in combination with ASSNAC, PFD, or ASSNAC + PFD (scale bars: 200 μm). Data are presented as mean ± SD (*n* = 3 independent experiments). * *p* < 0.05, ** *p* < 0.01, *** *p* < 0.001, **** *p* < 0.0001.

Excessive accumulation of ECM components, particularly collagens, is a hallmark of pulmonary fibrosis [47,48]. To evaluate the effect of ASSNAC on ECM deposition, we performed immunofluorescence staining for Collagen I and Collagen III in TGF-β1-stimulated cells. As shown in Figure 8a, TGF-β1 treatment resulted in a significant increase in red fluorescence intensity corresponding to Collagen I expression compared to the control group. Treatment with free PFD moderately reduced Collagen I levels, while ASSNAC showed a more pronounced inhibitory effect. Notably, the combination of ASSNAC + PFD led to the most substantial reduction in Collagen I expression. A similar trend was observed for Collagen III (Figure 8b), where the green fluorescence intensity was markedly elevated by TGF-β1 and significantly suppressed by the ASSNAC + PFD formulation. Quantitative analysis of the fluorescence intensity confirmed these observations.

To further validate the anti-fibrotic efficacy at the molecular level, we quantified the mRNA expression of key fibrotic markers using quantitative real-time PCR (qRT-PCR). As shown in Figure 7b, stimulation with TGF-β1 caused a drastic upregulation in the mRNA levels of all three markers compared to the control group, confirming the successful induction of the fibrotic phenotype. While treatment with ASSNAC or PFD alone resulted in a moderate reduction in gene expression, the combined treatment with ASSNAC + PFD exhibited the most potent inhibitory effect. The combination group showed a statistically significant decrease in the mRNA levels of Collagen I, Collagen III, and α-SMA compared to the monotherapy groups. These findings demonstrated that ASSNAC combined with PFD exerted anti-fibrotic effects and inhibited profibrotic gene transcription. Consistent with immunofluorescence results, the dual-drug regimen interferes with fibrotic progression more thoroughly than monotherapy.

### 2.4. Therapeutic Efficacy in BLM-Induced Pulmonary Fibrosis In Vivo

#### 2.4.1. Attenuation of Histopathological Damage and Collagen Deposition

**Figure 9:**
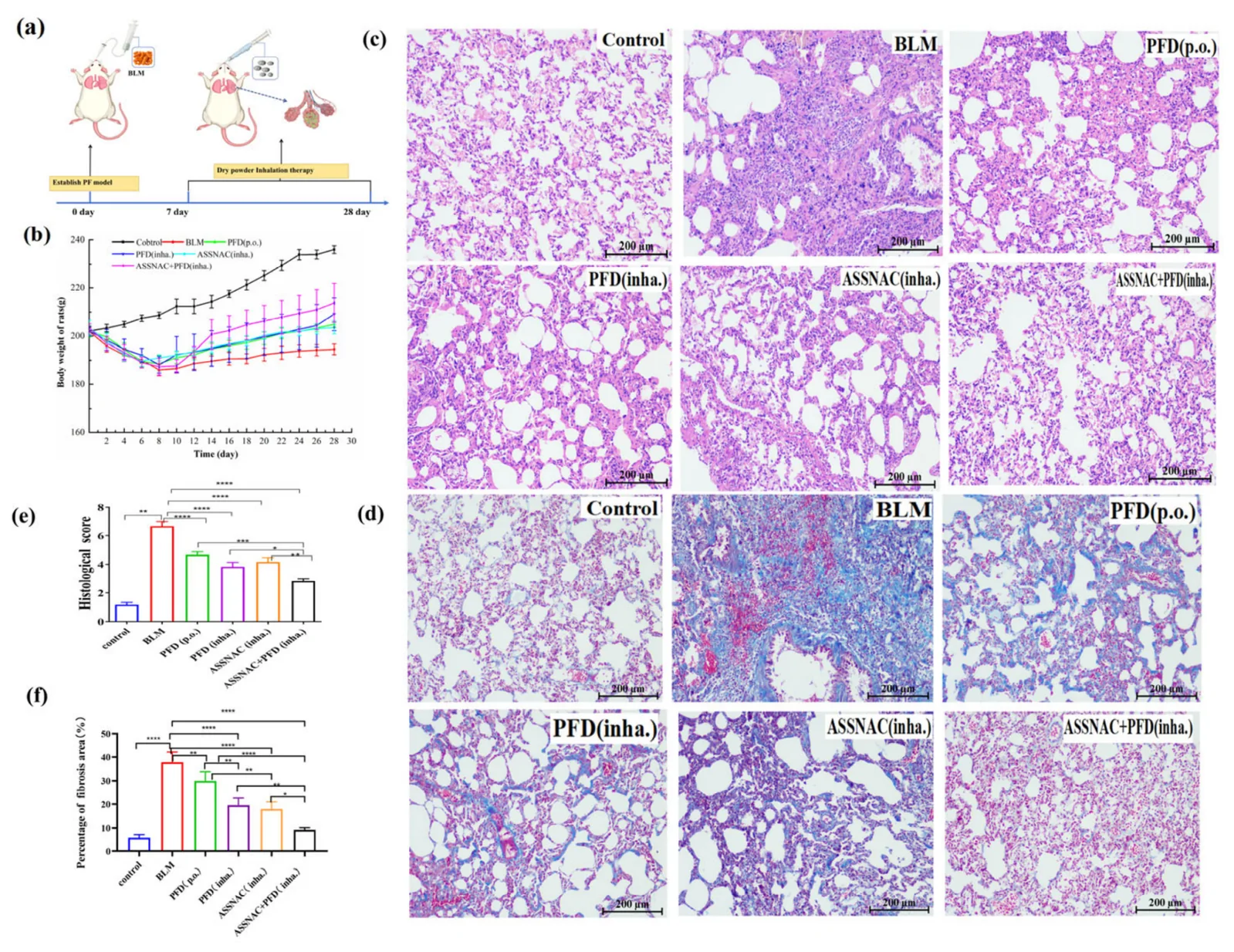
The therapeutic efficacy of ASSNAC + PFD/DPIs in the BLM-induced PF model in vivo. (**a**) Schematic of the treatment regimens. (**b**) Body weight changes in rats during treatment. (**c**) Representative H&E staining of lung tissues from different groups (scale bars: 200 μm). (**d**) Representative Masson’s trichrome staining (scale bars: 200 μm). (**e**,**f**) The modified Ashcroft score and quantitative analysis of fibrotic areas. All data are expressed as mean ± SD (*n* = 3 independent experiments). * *p* < 0.05, ** *p* < 0.01, *** *p* < 0.001, **** *p* < 0.0001.

The therapeutic efficacy of the combined strategy was evaluated in a BLM-induced PF rat model. Figure 9a illustrates the experimental timeline. Pulmonary fibrosis was induced by intratracheal instillation of BLM on day 0. From day 7 to day 28, rats received respective treatments via DPIs or oral administration.

Body weight changes were recorded to assess systemic toxicity and general health status of rats during the whole experiment (Figure 9b). Obvious weight loss occurred in the BLM model group within the first 8 days post-administration, followed by slow and inadequate weight recovery for the remaining duration. Oral PFD and single-agent inhalation treatments partially facilitated weight regain. The ASSNAC + PFD (inha.) group exhibited the optimal body weight recovery. These results demonstrated that combination inhalation ameliorated BLM-induced systemic cachexia and improved physical conditions in fibrotic rats.

Figure 9c presented the histopathological analysis via H&E staining. The BLM group exhibited severe architectural distortion, alveolar collapse, and massive inflammatory infiltration compared to the intact structure of the control group. While oral PFD and monotherapies provided moderate protection, the ASSNAC + PFD (inha.) group demonstrated the most significant preservation of alveolar integrity, markedly reducing both inflammation and tissue destruction.

To specifically assess fibrosis progression, Masson’s trichrome staining was performed for histological observation, accompanied by quantitative calculation of collagen fibrotic area and modified Ashcroft pathological scoring (Figure 9d–f). The BLM group showed extensive blue staining, indicating severe collagen deposition. Quantitative analysis and modified Ashcroft pathological scoring revealed that oral PFD and single-agent inhalation groups reduced fibrosis to varying degrees, whereas the ASSNAC + PFD combination achieved the lowest percentage of fibrotic area and the modified Ashcroft score. This highlights the combined therapy advantage: the antioxidant capacity of ASSNAC complements the pharmacological effect of PFD, offering superior structural protection against PF compared to monotherapy or oral administration.

#### 2.4.2. Study on Anti-Fibrotic Protein and Gene Expression

**Figure 10:**
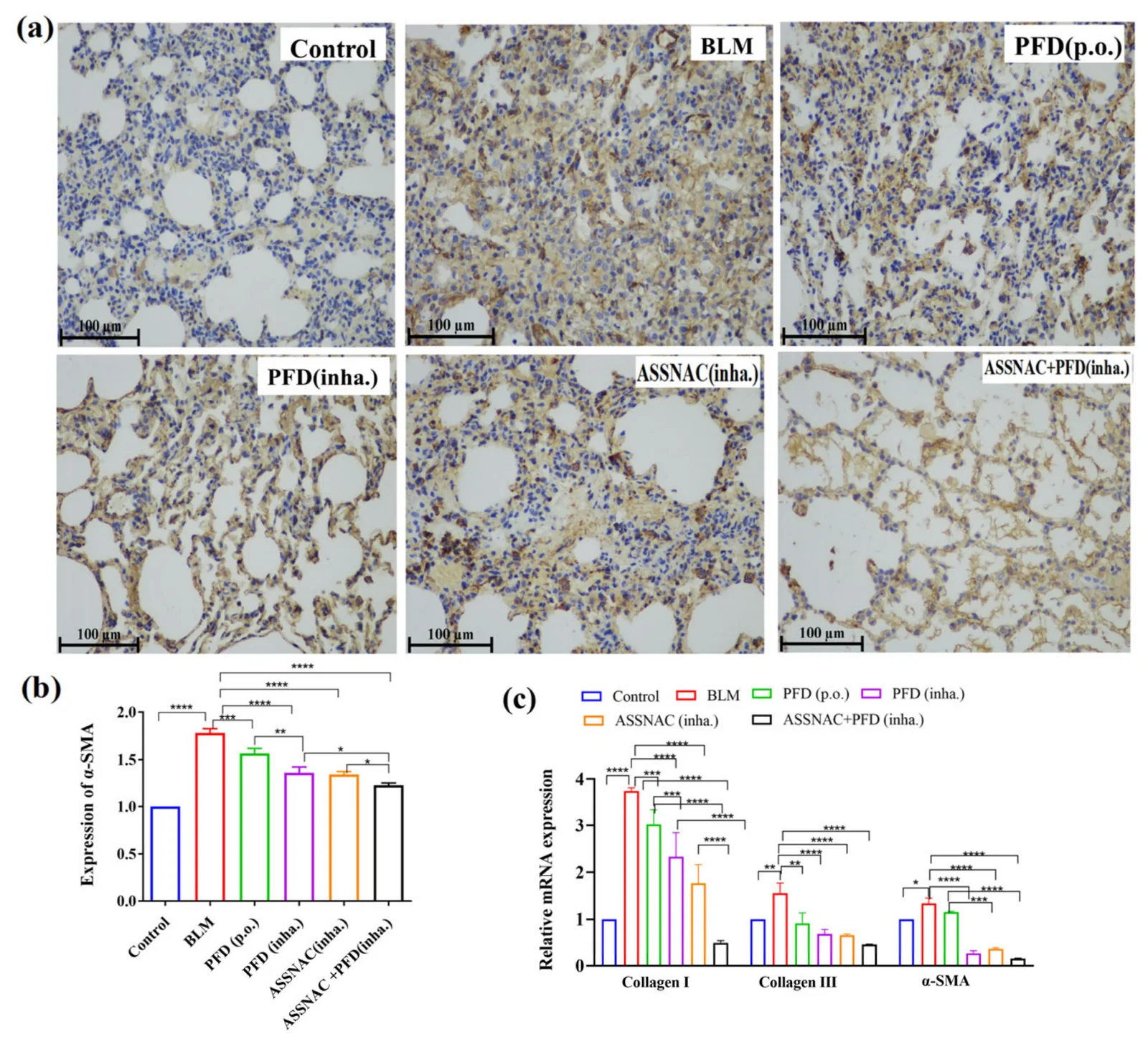
Expression of fibrosis markers. (**a**) Immunohistochemical staining of α-SMA in lung tissues (scale bars: 200 μm). (**b**) Quantitative analysis of α-SMA-positive area. (**c**) Relative mRNA expression levels of Collagen I, Collagen III, and α-SMA in lung tissues. All data are shown as the mean ± SD (*n* = 3 independent experiments). * *p* < 0.05, ** *p* < 0.01, *** *p* < 0.001, **** *p* < 0.0001.

Immunohistochemical staining (Figure 10a,b) and qRT-PCR analysis (Figure 10c) were performed to evaluate the expression of fibrotic markers at both protein and transcriptional levels. The BLM group exhibited intense positive staining and markedly upregulated mRNA expression of Collagen I, Collagen III, and α-SMA, confirming active fibrogenesis. Notably, inhaled PFD/DIPs (1.5 mg/kg) achieved comparable downregulation of these fibrotic markers to oral PFD at a 20-fold higher dose (30 mg/kg), demonstrating that the inhaled administration, even at a substantially reduced dose, effectively delivers PFD to the target site and suppresses fibrosis. Encouragingly, the ASSNAC + PFD/DIPs group further significantly reduced the levels of fibrotic markers compared to single-drug groups. Collectively, these results demonstrate that co-delivery of ASSNAC + PFD via inhalation achieves superior anti-fibrotic efficacy.

### 2.5. Investigation into Antioxidant and Anti-Fibrotic Mechanisms

#### 2.5.1. Restoration of Redox Homeostasis and Suppression of Oxidative Stress

**Figure 11:**
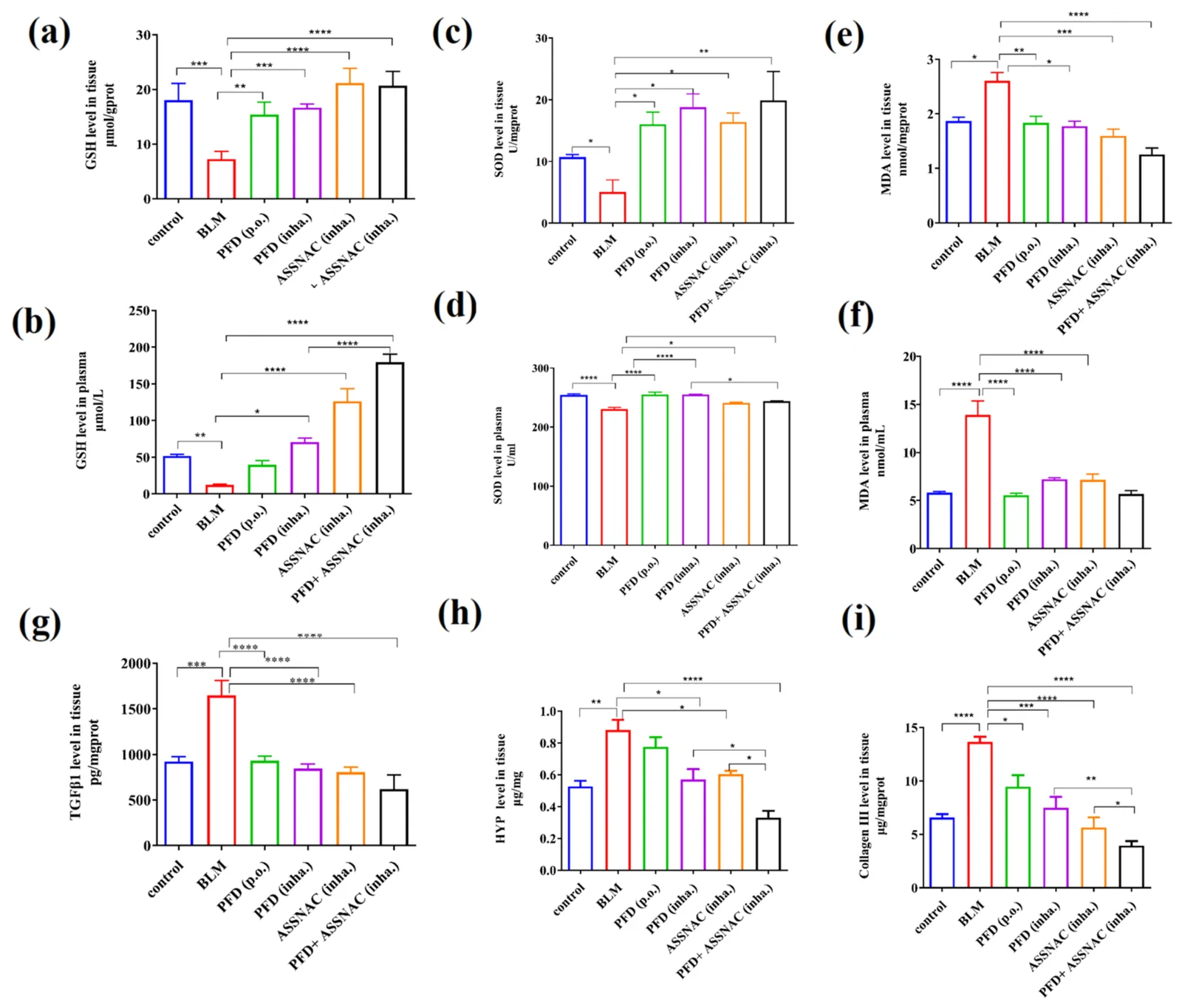
Levels of oxidative stress markers and fibrosis-related proteins in lung tissue and plasma. (**a**–**f**) Levels of GSH, SOD, and MDA in lung tissue and plasma. (**g**–**i**) Levels of TGF-β1, HYP, and Collagen III in lung tissue. All data are presented as mean ± SD (*n* = 3 independent experiments). * *p* < 0.05, ** *p* < 0.01, *** *p* < 0.001, **** *p* < 0.0001.

Oxidative stress plays a pivotal role in the pathogenesis of pulmonary fibrosis [49]. We evaluated the antioxidant efficacy by measuring GSH, SOD, and MDA levels in both lung tissue and plasma. As shown in Figure 11a,b, the BLM group exhibited a significant depletion of GSH levels in both tissue and plasma compared to the control group, indicating severe oxidative damage. While oral PFD showed limited recovery, the ASSNAC + PFD (inha.) group demonstrated the most substantial restoration of GSH levels, significantly surpassing the monotherapy groups. A similar trend was observed for SOD activity (Figure 11c,d); the combination therapy effectively reversed the BLM-induced reduction in SOD activity, suggesting a potent enhancement of the endogenous antioxidant defense system.

Conversely, MDA, a marker of lipid peroxidation, was significantly elevated in the BLM group (Figure 11e,f). Both tissue and plasma MDA levels were markedly reduced in the ASSNAC + PFD (inha.) group, showing the lowest levels among all treatment groups. These results indicated that the combined effect of ASSNAC and PFD effectively scavenges ROS and alleviates oxidative stress, creating a favorable microenvironment for tissue repair.

#### 2.5.2. Downregulation of TGF-β1 Signaling and ECM Deposition

The TGF-β1 pathway is the canonical driver of fibrosis. We investigated whether the therapeutic benefits were mediated through the inhibition of this pathway. Figure 11g showed that TGF-β1 levels were significantly upregulated in the BLM group. Treatment with ASSNAC + PFD (inha.) resulted in the most pronounced reduction in TGF-β1 expression, effectively suppressing the upstream fibrotic signaling.

To confirm the downstream effects on ECM accumulation, we measured HYP content and Collagen III levels. As illustrated in Figure 11h,i, the combination therapy significantly decreased HYP and Collagen III levels compared to the BLM group and monotherapy groups, further corroborating the histological findings of reduced fibrosis.

### 2.6. Systemic Safety and Toxicity Profile

**Figure 12:**
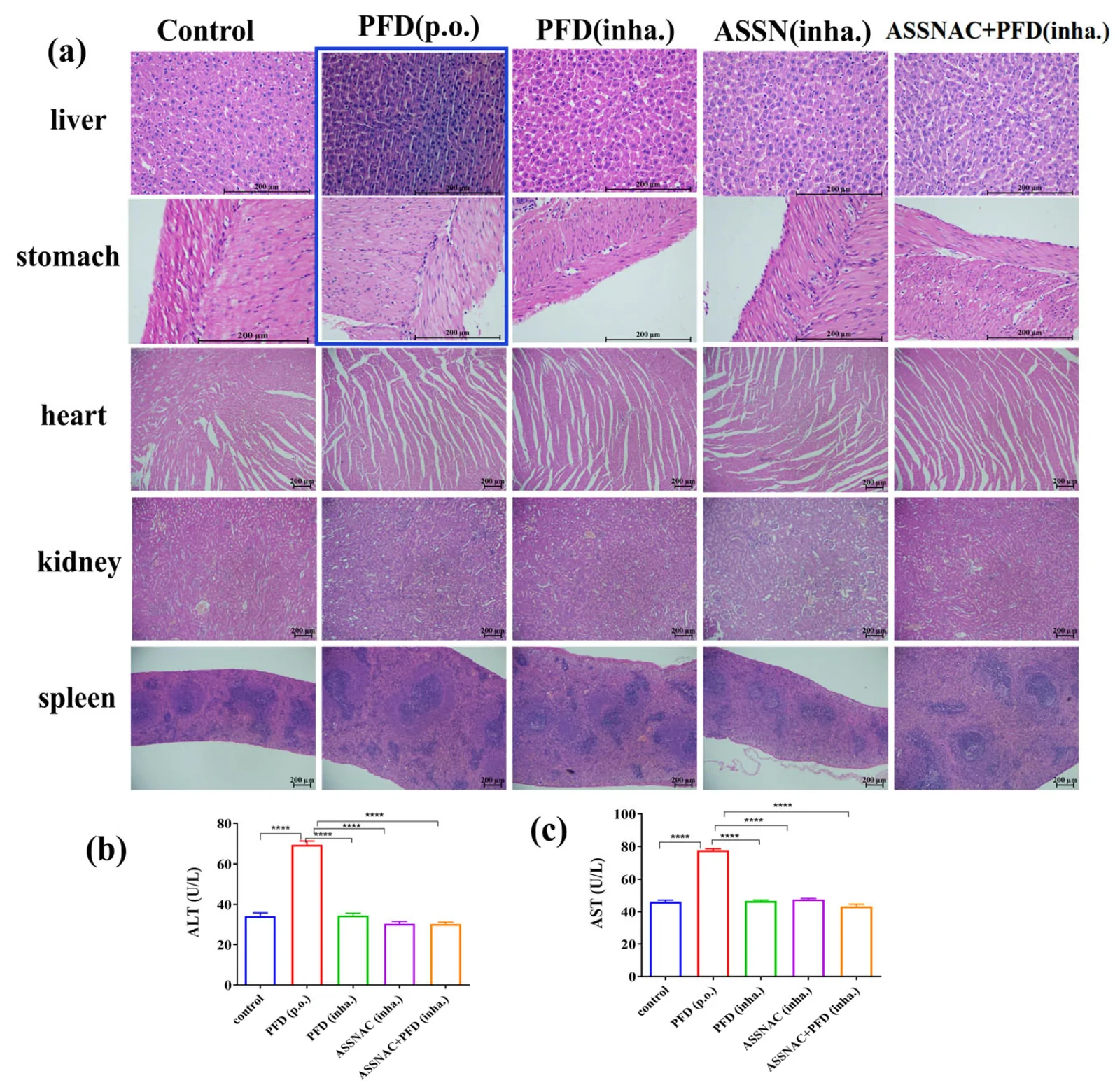
The toxicity effects of different administration methods. (**a**) H&E staining of the liver, stomach, heart, kidney, and spleen of the rats. Group settings: blank control, oral PFD, inhaled PFD/DPIs, inhaled ASSNAC/DPIs, inhaled ASSNAC + PFD/DPIs (Scale: 200 μm). (**b**,**c**) The levels of ALT and AST in plasma. All data are expressed as mean ± SD (*n* = 3 independent experiments). **** *p* < 0.0001.

To evaluate systemic safety, major organs were stained with H&E (Figure 12a). Alanine Aminotransferase (ALT) (Figure 12b) and Aspartate Aminotransferase (AST) (Figure 12c) in plasma were tested to evaluate liver biochemistry. Oral PFD triggered apparent liver and gastric pathological damage (as highlighted in the blue box of the Figure 12a), along with elevated ALT and AST, which validated hepatic toxicity. All inhalation groups exhibited regular tissue microstructure consistent with the control group, with normal ALT and AST levels and no pathological abnormalities in the heart, kidney and spleen. This inhalation delivery avoids systemic toxicity of oral PFD. Direct pulmonary drug delivery lessens undesired organ exposure, alleviates liver and gastric injury and improves local therapeutic effect. The co-delivery system possesses better safety than oral medication.

## 3. Conclusions

This study presents a promising strategy for the treatment of pulmonary fibrosis through the development of an inhalable, co-loaded liposomal dry powder. A key feature of this work is the utilization of a low-temperature spray-freeze-drying technique. This method, coupled with the strategic incorporation of L-leucine, yielded a formulation with optimized physicochemical properties, specifically characterized by reduced hygroscopicity and enhanced flowability, thereby ensuring efficient aerosolization and deep pulmonary deposition.

Furthermore, the co-delivery system capitalizes on a combined antioxidant mechanism, in which ASSNAC and PFD complement each other to combat oxidative stress more effectively than monotherapy. Importantly, inhalation delivery of the ASSNAC + PFD/DPIs achieves superior anti-fibrotic efficacy at a fraction of the oral dose (20-fold lower), highlighting the profound advantage of direct lung targeting in maximizing local bioavailability while minimizing systemic exposure. Consequently, the ASSNAC + PFD dry powder inhaler represents a superior alternative to conventional oral administration, offering an efficacious and safer therapeutic modality for PF.

## 4. Materials and Methods

### 4.1. Materials

ASSNAC (purity ≥ 96%) was synthesized in our laboratory based on earlier reports. PFD was sourced from Beijing Continent Pharmaceuticals Co., Ltd. (Etuary^®^; Beijing, China), while the PFD active pharmaceutical ingredient was procured from Aladdin Biochemical Technology Co., Ltd. (Shanghai, China). Cell culture reagents including RPMI-1640, F12K media, and fetal bovine serum (FBS) were obtained from Biological Industries (Beit Haemek, Israel). Human fetal lung fibroblast (HFL-1) cells were authenticated from the BeNa Culture Collection (ATCC origin; Langfang, China). Beas-2B lung epithelial cells were kindly provided by the School of Basic Medical Sciences, Shandong University (Jinan, China). Key biochemical assay kits (GSH, MDA, SOD, HYP, AST, ALT) were sourced from Nanjing Jiancheng Bioengineering Institute (Nanjing, China) and PeproTech (Cranbury, NJ, USA), respectively. Liposomal components (lecithin, cholesterol, DSPE-PEG 2000) and coumarin 6 were supplied by Aladdin Reagent Co., Ltd. (Shanghai, China) and Solarbio Co., Ltd. (Beijing, China). Molecular biology reagents including SYBR^®^ Green PCR Master Mix (TOYOBO Co., Ltd., Osaka, Japan), RNA extraction kits (Invitrogen, Carlsbad, CA, USA), and primers (Beijing Dingguo Changsheng Biotechnology Co., Ltd., Beijing, China), were used as specified. Antibodies against Collagen I, Collagen III and α-SMA were received from Cell Signaling Technologies (Danvers, MA, USA). Lactose, sucrose, mannitol, and L-leucine were supplied by Beijing Fengli Jingqiu Pharmaceutical Co., Ltd. (Beijing, China). Bleomycin (BLM) was acquired from Shanghai Yuanye Biology Co., Ltd. (Shanghai, China). All other chemicals used were of analytical grade.

### 4.2. Preparation of ASSNAC + PFD Co-Loaded Liposomes (ASSNAC + PFD/Lip) and DPIs

#### 4.2.1. Preparation of ASSNAC + PFD/Lip

ASSNAC and PFD co-loaded liposomes were prepared using a calcium acetate gradient active loading method. Blank liposomes (phospholipids/cholesterol/DSPE-PEG 2000) were formed via a water-in-oil emulsion, followed by solvent removal, sonication, filtration, and dialysis against sodium sulfate to generate the gradient. Drugs were added and incubated at 40 °C for 60 min.

#### 4.2.2. Preparation of ASSNAC + PFD/Lip DPIs (ASSNAC + PFD/DPIs)

DPIs were prepared using a spray-freeze-drying process. A mixed solution containing ASSNAC and PFD co-loaded liposomes and freeze-drying protective excipients (lactose, sucrose, mannitol, L-leucine) was prepared in a co-solvent system. This solution was atomized through a nozzle directly into a reservoir of liquid nitrogen, leading to the instantaneous vitrification of fine droplets. The frozen microparticles were then collected and subjected to lyophilization. Finally, a light, porous powder suitable for pulmonary delivery was obtained. The excipient composition of DPIs is presented in Table 2.

### 4.3. Characterization of Liposomes and DPIs

#### 4.3.1. Drug Loading (DL) and Encapsulation Efficiency (EE)

The liposome samples were transferred into the ultrafiltration centrifugal tube and centrifuged for 15 min at 3000 rpm. The filtrates were diluted with methanol before assaying by HPLC for free PFD and ASSNAC. The co-loaded liposomes were taken and dissolved in methanol. The contents of PFD and ASSNAC in the above samples were determined by HPLC as the total amount of PFD and ASSNAC. The DL and EE of ASSNAC were calculated according to the following Equations 1 and 2:

(1) $$\mathrm{EE}\;\%=\frac{\;\mathrm{Total}\;\mathrm{drug}\;\mathrm{amount}-\mathrm{free}\;\mathrm{drug}\;\mathrm{amount}}{\;\mathrm{Total}\;\mathrm{drug}\;\mathrm{amount}\;}\times 100\%$$

(2) $$\mathrm{DL}\;\%=\frac{\;\mathrm{Total}\;\mathrm{drug}\;\mathrm{amount}-\mathrm{free}\;\mathrm{drug}\;\mathrm{amount}\;}{\mathrm{Total}\;\mathrm{liposomes}\;\mathrm{amount}\;}\times 100\%$$

#### 4.3.2. Particle Size, PDI, Zeta Potential of ASSNAC + PFD/Lip

Liposome samples were diluted 5-fold with PBS (pH 7.4) to an appropriate concentration for measurement. The particle size, PDI, and zeta potential of ASSNAC + PFD/Lip before freeze-drying and after re-dissolution of the spray-freeze-dried powder were determined using dynamic light scattering (DLS) (Malvern Panalytical, Malvern, UK).

#### 4.3.3. Cryo-Transmission Electron Microscopy (Cryo-TEM)

The liposome dispersion was diluted 5-fold with PBS. A 3 μL aliquot of the liposome sample was promptly deposited onto the copper grid, and the surplus liquid was carefully blotted with filter paper. The grid was then swiftly immersed in liquid ethane for rapid freezing and transferred into liquid nitrogen. The morphology of ASSNAC + PFD co-loaded liposomes was observed using a cryo-electron microscope (Thermo Fisher Scientific, Waltham, MA, USA) at cryogenic temperature.

#### 4.3.4. Release Experiments In Vitro

To simulate the acidic fibrotic microenvironment and the normal lung milieu, drug release was assessed at pH 5.5 and 7.4. The API suspensions and liposomes (1.5 mL) were placed into dialysis bags and incubated in the release medium (pH 5.5 or pH 7.4 PBS containing 0.2% Tween 80, 30 mL) at 37 °C with shaking at 100 rpm. Samples were collected at predetermined time intervals (0.08, 0.17, 0.25, 0.33, 0.5, 0.75, 1, 1.5, 2, 3, 4, 6, 8, 10, and 12 h), with 0.5 mL aliquots withdrawn at each time point. To ensure the integrity of the release environment, an equivalent volume (0.5 mL) of fresh release medium was immediately added after each sampling. The collected samples were then filtered through appropriate membranes to remove any particulate matter, and the filtrates were analyzed using HPLC for accurate quantification of the released compounds. The cumulative release data were fitted to kinetic models using Origin 8.5, and the best-fit model was selected based on the fitting parameters to elucidate the underlying release mechanism.

#### 4.3.5. Scanning Electron Microscopy

Sample morphology was analyzed using a scanning electron microscope (Hitachi High-Technologies Corporation, Tokyo, Japan). Powder samples were uniformly distributed onto conductive carbon tape affixed to aluminum stubs. To improve conductivity, the samples were sputter-coated with a thin layer of a gold–palladium alloy, approximately 10–15 nm in thickness. Imaging was conducted under high vacuum at accelerating voltages ranging from 5 to 20 kV to capture representative surface features.

#### 4.3.6. Thermogravimetric (TG) and Derivative Thermogravimetric (DTG) Analysis

The thermal stability of DPI formulations F4, F5, and F6 was characterized by TG and DTG (TA Instruments, New Castle, DE, USA) analysis. Approximately 3–5 mg of powder sample was accurately weighed and placed into an alumina crucible. Samples were heated from room temperature up to 300 °C at a constant heating rate of 10 °C/min under continuous nitrogen purge (flow rate: 20 mL/min). The real-time mass variation in each formulation was automatically recorded against temperature. Thermal decomposition temperature, moisture loss and thermal degradation behavior were compared among F4, F5 and F6. DTG curves were derived to determine the characteristic peak temperatures (T_max_) of each degradation stage, and the corresponding weight-loss percentages (Δm) were further calculated.

#### 4.3.7. Differential Scanning Calorimetry (DSC)

Thermal properties were assessed using DSC (DSC Q200, TA Instruments, USA). Before testing, the instrument was calibrated with indium to ensure accuracy in temperature and enthalpy measurements. Samples weighing approximately 3–5 mg were enclosed in aluminum pans and subjected to heating from 25 °C to 300 °C at a constant rate of 10 °C/min in a nitrogen atmosphere. Heat flow data were collected to identify thermal transitions.

#### 4.3.8. X-Ray Diffractometer (XRD)

Powder samples’ crystalline properties were assessed via X-ray diffraction (MiniFlex 600, Rigaku, Tokyo, Japan) at 40 kV and 15 mA using Cu Kα radiation. The samples were placed on a glass holder and scanned from 5° to 35° at a step size of 0.02° and a scanning speed of 3°/min. The distinctive diffraction peaks were matched with standard reference patterns to validate the crystalline phases in the formulations.

### 4.4. Aerodynamic Particle Size Analysis

The aerosol performance was assessed using a Next-Generation Impactor (NGI) (Copley Scientific, Nottingham, UK) at a flow rate of 60 L/min (4 s actuation) in vitro. Powder samples (5–10 mg) were loaded into size 3 HPMC capsules and subsequently aerosolised using a high-resistance dry powder inhaler connected to the NGI via a USP induction port. The deposition of the pharmaceutical compound was conducted on the induction port, the inhaler, and each NGI stage. The recovery of these deposits was achieved through the utilization of a water/acetonitrile mixture, followed by quantitative analysis via HPLC. Aerodynamic parameters were calculated, including the mass median aerodynamic diameter (MMAD), geometric standard deviation (GSD), and fine particle fraction (FPF, <5 μm).

### 4.5. Dynamic Vapor Sorption (DVS)

The hygroscopicity of the engineered dry powder formulations was evaluated using a DVS analyzer (DVS-Intrinsic, Surface Measurement Systems Ltd., London, UK) at 25.0 ± 0.1 °C. Approximately 10–15 mg of sample was dried under 0% RH until a stable mass baseline was reached, followed by a stepwise increase in relative humidity from 0% to 90% in 10% increments. After the adsorption cycle, a desorption profile was generated by reducing RH from 90% back to 0%. The mass change at each equilibrium point was recorded to construct sorption–desorption isotherms for assessing formulation hygroscopicity and physical stability.

### 4.6. Cellular Uptake Studies of Liposomes In Vitro

The cellular uptake of coumarin 6 and rhodamine B liposomes by Beas-2B and HFL-1 was investigated. HFL-1 or Beas-2B cells were seeded into 24-well cell culture plates at a density of 1 × 10^5^ cells/mL and incubated for 24 h under standard conditions (37 °C, 5% CO_2_). Following incubation, the medium was replaced with fresh serum-free medium containing either coumarin 6-labeled liposomes or rhodamine B-labeled liposomes. After incubating the cells for 15, 30, 60 and 120 min, the medium was discarded, washed with PBS three times, stained with DAPI and fixed with paraformaldehyde. Finally, the cells were observed under a microscope for fluorescence distribution. Images were captured using a microscope (IX71, Olympus Corporation, Tokyo, Japan).

### 4.7. Hemolysis Study of DPIs In Vitro

The erythrocyte suspension (concentration: 2%) was prepared as follows: Rat venous blood samples were collected and centrifuged at 2000 rpm for 10 min to separate the plasma. Following plasma removal, the remaining blood cells were carefully resuspended in chilled saline solution to obtain the erythrocyte suspension for subsequent experiments.

Various concentrations of ASSNAC + PFD/DPIs were added to the erythrocyte suspension. The concentrations of ASSNAC ranged from 10 to 50 μg/mL, and the concentrations of PFD ranged from 8 to 40 μg/mL. The positive control was prepared by replacing saline with water in erythrocyte suspension, and the negative control was erythrocyte suspension without drugs. All samples were incubated at 37 °C for 1 h, followed by centrifugation. The absorbance of the resulting supernatant was then measured at 540 nm, and the hemolysis rate was calculated using Equation (3).

(3) $$\mathrm{Hemolysis}\;\mathrm{rate}\;(\%)=\frac{{\mathrm{OD}}_{\mathrm{test}\;\mathrm{group}}-{\mathrm{OD}}_{\mathrm{negative}\;\mathrm{control}}}{{\mathrm{OD}}_{\mathrm{positive}\;\mathrm{control}}-{\mathrm{OD}}_{\mathrm{negative}\;\mathrm{control}}}$$

### 4.8. Efficacy of ASSNAC + PFD/Lip for PF In Vitro

#### 4.8.1. Cell Cultures

Beas-2B cells were maintained in 1640 medium, while HFL-1 cells were cultured in F12K medium. Both media were enriched with 10% FBS, and the cells were incubated at 37 °C in 5% CO_2_.

#### 4.8.2. MTT Assay

The effects of varying concentrations of blank DPIs, ASSNAC/DPIs, PFD/DPIs, and ASSNAC + PFD/DPIs on the viability of HFL-1 and Beas-2B cells were evaluated using the MTT assay. HFL-1 or Beas-2B cells were seeded into 96-well plates at a density of 1 × 10^5^ cells/mL and cultured at 37 °C in 5% CO_2_ for 24 h.

Cells were incubated for 48 h with different concentrations of blank DPIs, ASSNAC/DPIs (0, 30, 60, 120, and 240 μM), PFD/DPIs (0, 50, 100, 200, and 400 μM), and ASSNAC + PFD/DPIs (ASSNAC: 0, 30, 60, 120, and 240 μM; PFD: 0, 50, 100, 200, and 400 μM). After the incubation period, 20 μL of MTT solution was added to each well and allowed to incubate for 4 h. Following this, the medium was carefully removed from the 96-well plates, and 150 μL of DMSO solution was added to each well to dissolve the formazan crystals. The plates were then gently shaken for 15 min to ensure complete dissolution. The optical density of the resulting solution was measured at 490 nm using an enzyme-linked immunosorbent assay reader (Infinite® M200 PRO, TECAN, Männedorf, Switzerland). Finally, cell viability was determined based on the calculations derived from Equation (4).

(4) $$\mathrm{Cells}\;\mathrm{viability}=\frac{{\mathrm{OD}}_{\mathrm{test}}-{\mathrm{OD}}_{\mathrm{blank}}}{{\mathrm{OD}}_{\mathrm{control}}-{\mathrm{OD}}_{\mathrm{blank}}}\times 100\%$$

#### 4.8.3. Analysis of Beas-2B Cell Morphology

Beas-2B cells were seeded in 6-well dishes, exposed to TGF-β1 (5 ng/mL) for 24 h to induce fibrotic morphological changes, and then treated with ASSNAC, PFD, or their combination for another 24 h. Subsequently, morphological changes were examined using phase-contrast microscopy, and images were captured.

#### 4.8.4. Migration Assay in Beas-2B Cells

Confluent Beas-2B cell monolayers were scratched, treated with TGF-β1 and the specified compounds in 1% FBS medium, and wound closure was monitored at 0 and 48 h post-scratch using an inverted microscope. Quantification was performed using ImageJ. 1.46r software.

#### 4.8.5. Invasion Assay in HFL-1 Cells

Migration of HFL-1 cells was evaluated utilizing Transwell chambers with 8 μm pores. Cells in serum-free medium were placed in the upper wells, while the lower wells contained 10% FBS medium with TGF-β1 and test substances. After 24 h, migrated cells on the lower membrane were fixed, stained with crystal violet, imaged using an inverted microscope, and counted in five random fields per well.

#### 4.8.6. The mRNA Expressions of Collagen I, Collagen III, and α-SMA in Beas-2B Cells

The in vitro modeling and dosing regimen were consistent with those described in Section 4.8.3. Cells were harvested for mRNA expression of Collagen I, Collagen III, and α-SMA. Extraction and reverse transcription of RNAs and gene amplification of Collagen I, Collagen III, and α-SMA were performed according to the conditions described in the Supporting Information. Collagen I, Collagen III, and α-SMA and GAPDH primers used for PCR are listed in Table 3.

#### 4.8.7. The Expression of Collagen I and Collagen III Proteins in Beas-2B Cells Examined by Immunofluorescence

The method for the establishment of the PF model in vitro was the same as described in Section 4.8.3. The study groups were set as follows: control, TGF-β1, ASSNAC/DPIs (90 μM), PFD/DPIs (150 μM), and ASSNAC + PFD/DPIs (90 μM, 150 μM) groups. Cells were washed three times with PBS and fixed with 4% paraformaldehyde for 15 min at the end of the culture. The cells were first blocked with sheep serum (1:100) and subsequently incubated with primary antibodies overnight at 4 °C. Following this, the cells were treated with secondary antibodies for 1 h at room temperature, then stained with DAPI for 5 min to visualize nuclei. After each step, the cells were thoroughly washed three times with PBS to remove any unbound reagents. Fluorescence images were acquired using an epifluorescence microscope (IX71, Olympus, Japan). The primary antibodies employed included anti-Collagen I and anti-Collagen III.

### 4.9. Pharmacodynamic Research of ASSNAC + PFD Co-Loaded Liposomes In Vivo

#### 4.9.1. Experimental Animals

Adult male Sprague–Dawley rats (8–9 weeks, 200 ± 20 g) were purchased from Jinan Pengyue Experimental Animal Breeding Co., Ltd. (Jinan, China). All rats were maintained at 20–25 °C and 45–60% RH with a 12 h day and night cycle. To ensure the rats were well-accustomed to their new surroundings, they were provided with unrestricted access to water and food for a period of 7 days prior to the commencement of the experiment. All experimental protocols were conducted in strict compliance with ethical standards and were formally approved by the Animal Care and Use Committee of Liaocheng University (Approval No. 2024061240, Liaocheng, Shandong, China).

#### 4.9.2. BLM-Induced PF Model and Drug Administration

The rat model of pulmonary fibrosis was established through endotracheal administration of BLM [45,46]. The rats were randomly divided into six groups (*n* = 6 per group): control, BLM-induced model (5 mg/kg), oral PFD, inhaled PFD/DPIs, inhaled ASSNAC/DPIs, and inhaled ASSNAC + PFD/DPIs. On day 0, all experimental groups except the control group underwent endotracheal infusion of BLM (5 mg/kg) to establish the pulmonary fibrosis model. Following a 7-day induction period, the therapeutic interventions were administered continuously for 21 days. Rat body weights were documented every other day during treatment. The oral PFD group received the PFD marketed products (30 mg/kg). The inhalation groups, including PFD/DPIs (1.5 mg/kg), ASSNAC/DPIs (1 mg/kg), and ASSNAC + PFD/DPIs (1 mg/kg, 1.5 mg/kg), were administered via dry powder inhalation once daily [19]. Meanwhile, the control and model groups received an equivalent volume of saline. On the 28th day, all rats were euthanized, and their plasma and lung tissues were collected for subsequent analysis. For all histological and biochemical analyses, 3 rats per group were randomly selected from the 6 rats per group, owing to limited kit capacity and sample volume.

#### 4.9.3. Histopathology

To meticulously examine the morphological changes within the lung tissues, the right lung was carefully immersed in a 4% paraformaldehyde solution for 24 h to ensure optimal fixation. Following this, the lung tissue was precisely sectioned into 5 μm thick slices. These sections were then subjected to staining using both hematoxylin and eosin (H&E) for general histological analysis and Masson’s trichrome for the assessment of collagen deposition and fibrosis. The stained tissue samples were meticulously examined and captured under a high-resolution microscope (Eclipse, Nikon Corporation, Tokyo, Japan) at 400× magnification. To quantify the fibrotic areas, Masson’s trichrome-stained sections were analyzed using Image J software, ensuring precise and reliable measurements. Lung histopathology was graded via the standardized modified Ashcroft scoring system under microscopic examination [50,51].

#### 4.9.4. Immunohistochemical Analysis

The expression of α-SMA in lung tissue samples was meticulously quantified through immunohistochemical analysis. Tissue sections underwent a series of preprocessing steps, including deparaffinization with xylene, rehydration, antigen retrieval, and blocking of nonspecific binding sites by incubation with 1% goat serum at 37 °C for 30 min. Following the blocking step, the sections were incubated overnight at 4 °C with a primary antibody targeting α-SMA. Subsequently, the samples were treated with an HRP-conjugated goat secondary antibody for 30 min, and the presence of α-SMA was visualized using a DAB detection kit. Finally, the stained tissue sections were carefully examined under a microscope at 400× magnification for detailed analysis.

#### 4.9.5. Determination of TGF-β1, HYP and Collagen III in Lung Tissues

A total of 30 mg of lung tissue was carefully weighed and transferred into a sterile container. Under ice bath conditions, 270 μL of sterile saline was added to the tissue. The mixture was then thoroughly ground in a sterile environment to prepare a 10% tissue homogenate. Following homogenization, the sample was centrifuged at 3500 rpm for 10 min at 4 °C. The resulting supernatant was carefully collected and transferred to a new container for subsequent analysis. The concentrations of TGF-β1, HYP, and Collagen III were measured using commercially available assay kits.

#### 4.9.6. mRNA Expression of Collagen I, Collagen III, and α-SMA in the Lung Tissue

A total of 30 mg of lung tissue was precisely weighed, and 1 mL of TRIzol was added to initiate the extraction process. The mixture was then incubated at room temperature for 5 min to ensure complete tissue decomposition. Subsequent steps, including RNA extraction, reverse transcription, and qPCR experiments, were meticulously carried out following the detailed protocols outlined in the Supporting Information. The primer sequences corresponding to each target gene are systematically presented in Table 2, with GAPDH employed as the reliable reference gene for normalization purposes.

#### 4.9.7. Determination of SOD, MDA and GSH in Plasma and Lung Tissues

Plasma preparation: Venous blood samples from rats were collected into heparinized EP tubes and centrifuged at 3000 rpm for 10 min at 4 °C. The plasma was carefully extracted and stored at −80 °C for further analysis.

Lung tissue homogenates were prepared following the protocol outlined in Section 4.9.5. Subsequently, the levels of SOD, MDA, and GSH in both plasma and lung tissue homogenates were quantified using commercially available assay kits, strictly adhering to the manufacturer’s instructions.

### 4.10. Safety Research In Vivo

Following drug administration, H&E staining was systematically performed on cardiac, hepatic, splenic, renal, and gastric tissues from rats across all experimental groups. Levels of ALT and AST in plasma were tested using commercial assay kits to evaluate hepatic function. The study groups included a control group, an oral PFD group, an inhaled PFD/DPIs group, an inhaled ASSNAC/DPIs group, and an inhaled ASSNAC + PFD/DPIs group. Histological studies paired with liver biochemical indices were employed to investigate the damage to each organ caused by the different drugs and administrations.

### 4.11. Statistical Analysis

All data are expressed as mean ± standard deviation (SD). Statistical comparisons were performed using one-way analysis of variance (ANOVA) followed by Tukey multiple comparison test. A value of *p* < 0.05 was considered statistically significant.

## Figures and Tables

**Table 1:** Thermal decomposition parameters of F4, F5, and F6 derived from TGA/DTG analysis.

F4			F5			F6		
T	Δm	T_max_	T	Δm	T_max_	T	Δm	T_max_
25–120	1.48	/	25–120	1.26	/	25–120	0.568	/
160–200	14.91	195.5	130–200	35.06	160.13	175–212	6.76	209.76
200–240	30.28	214.7	220–350	19.84	/	212–263	21.08	227.19
250–350	13.64	/	/	/	/	263–300	27.37	/

Note: T = temperature range of thermal decomposition (°C); Δm = mass-loss percentage (%); T_max_ = peak temperature of the maximum weight-loss rate on the DTG curve (°C).

**Table 2:** Excipient composition of DPIs (%, *w*/*v*).

Component	F1	F2	F3	F4	F5	F6
Sucrose	10	/	/	10	/	/
Lactose	/	10	/	/	10	/
Mannitol	/	/	10	/	/	10
L-Leucine	/	/	/	10	10	10

**Table 3:** Primer sequences used for qPCR analysis.

Gene	Forward	Reverse
α-SMA (human)	AGCCAAGCACTGTCAGGAATC	GTCACCCACGTAGCTGTCTT
Collagen I (human)	TTTGGATGGTGCCAAGGGAG	CACCATCATTTCCACGAGCA
Collagen III (human)	CGCCCTCCTAATGGTCAAGG	TTCTGAGGACCAGTAGGGCA
GAPDH (human)	AGAAGGCTGGGCTCATTTG	AGGGGCCATCCAGTCTTC
α-SMA (rat)	ACCATCGGGAATGAACGCTT	CTGTCAGCAATGCCTGGGTA
Collagen I (rat)	GATGGACTCAACGGTCTCCC	CGGCCACCATCTTGAGACTT
Collagen III (rat)	TGCAATGTGGGACCTGGTTT	GGGCAGTCTAGTGGCTCATC
GAPDH (rat)	GCACCGTCAAGGCTGAGAAC	TGGTGAAGACGCCAGTGGA

## Data Availability

The original contributions presented in this study are included in the article/Supplementary Material. Further inquiries can be directed to the corresponding author.

## Supplementary Materials

The following supporting information can be downloaded at: https://www.mdpi.com/article/10.3390/ph19091374/s1, Table S1: Encapsulation efficiency and drug loading of ASSNAC and PFD in the ASSNAC+PFD/Lip (mean ± SD, *n* = 3); Table S2: Aerodynamic parameters of spray freeze dried formulations F4, F5, and F6 (mean ± SD, *n* = 3); Table S3: Changes in particle characteristics of liposomes before and after dry powder reconstitution (mean ± SD, *n* = 3); Table S4: Kinetic-fitting parameters of drug release profiles for all formulations at pH 5.5; Table S5: Kinetic-fitting parameters of drug release profiles for all formulations at pH 7.4.
