# Supplementary material for: Development and Evaluation of a Novel Inhalable Liposomal Powder Co-Encapsulating ASSNAC and Pirfenidone via Spray Freeze-Drying for Targeted Pulmonary Fibrosis Therapy
Source: Pharmaceuticals (Basel). 2026 Aug 31;19(9):1374. doi: 10.3390/ph19091374 (PMC13610831; doi:10.3390/ph19091374)
Supplement: Supplementary file 1 [file pharmaceuticals-19-01374-s001.zip › pharmaceuticals-4424770-supplementary.pdf]

Results

Table S1. Encapsulation efficiency and drug loadingof ASSNAC and PFD in the ASSNAC+PFD/Lip (mean ± SD, n=3)

ASSNAC+PFD/Lip	EE%	DL (%)
ASSNAC	95.23 ± 2.35	9.52 ± 0.13
PFD	70.20 ± 2.90	5.50 ± 0.39

Table S2. Aerodynamic parameters of spray freeze dried formulations F4, F5, and F6 (mean ± SD, n=3)

	MMAD(μm)	GSD	FPF	ED (%)
F4	3.92 ± 0.37	2.03 ± 0.08	31.91 ± 3.31	95.18 ± 0.45
F5	3.02 ± 1.20	2.53 ± 0.30	24.97 ± 2.73	94.11 ± 4.00
F6	2.22 ± 0.11	1.98 ± 0.07	61.08 ± 2.45	94.08 ± 1.00

Table S3. Changes in particle characteristics of liposomes before and after dry powder reconstitution (mean ± SD, n=3)

	Original ASSNAC+PFD/Lip	ASSNAC+PFD/Lip after DPI reconstitution
Size (nm)	122.33 ± 1.53	125.67 ± 2.52
PDI	0.11 ± 0.01	0.12 ± 0.01
Zeta potential (mV)	-21.04 ± 0.62	-20.36 ± 0.10

Table S4. Kinetic-fitting parameters of drug release profiles for all formulations at pH 5.5

	Zero-order			First-order			Higuchi			Korsmeyer-Peppas			Weibull models					
	a	b	R^2	a	b	R^2	a	b	R^2	a	n	R^2	A	Xc	d	k	R^2
ASSNAC/API	8.47±2.14	36.26±3.89	0.51	84.88±4.62	1.72±0.83	0.93	32.78±4.42	19.13±3.93	0.79	55.16±2.01	0.32±0.03	0.88	100	0.03±0.02	0.61±0.04	1.04±0.04	0.99
PFD/API	4.3±0.99	60.50±6.50	0.55	96.72±1.94	2.29±0.24	0.94	18.81±2.83	45.47±6.32	0.75	67.75±2.88	0.19±0.02	0.88	100	0.03±0.02	0.61±0.04	1.04±0.04	0.99
ASSNAC/Lip	7.40±0.58	21.12±2.23	0.88	90.75±7.07	0.30±0.05	0.91	27.33±0.68	2.46±1.08	0.99	29.72±0.71	0.47±0.01	0.99	100	-0.80±0.24	1.05±0.10	0.19±0.01	0.98
PFD/Lip	5.67±0.74	40.56±3.72	0.8	81.46±2.65	0.77±0.09	0.91	22.97±1.52	21.97±2.98	0.94	46.08±1.04	0.29±0.01	0.98	100	0.01±0.02	0.53±0.02	0.41±0.01	0.99

Table S5. Kinetic-fitting parameters of drug release profiles for all formulations at pH 7.4

	Zero-order			First-order			Higuchi			Korsmeyer-Peppas			Weibull models				
	a	b	R^2	a	b	R^2	a	b	R^2	a	n	R^2	A	X_c	d	k	R^2
ASSNAC/API	5.95±1.44	43.81±6.75	0.53	96.16±1.93	2.03±0.13	0.98	25.21±3.94	29.11±6.29	0.74	60.33±3.48	0.25±0.03	0.85	100	-0.01±0.02	0.78±0.06	1.62±0.09	0.99
PFD/API	5.05±1.47	57.92±7.02	0.43	96.63±2.38	1.79±0.18	0.95	22.93±4.14	40.35±7.20	0.67	67.10±3.30	0.22±0.03	0.83	100	-0.07±0.04	0.83±0.07	1.37±0.11	0.99
ASSNAC/Lip	7.14±0.36	10.09±2.06	0.87	95.51±4.39	0.18±0.02	0.99	26.97±0.34	6.46±0.67	0.99	19.65±0.52	0.60±0.01	0.99	100	-0.35±0.08	1.04±0.04	0.15±0.01	0.99
PFD/Lip	4.39±0.68	52.89±4.29	0.74	89.52±2.21	1.07±0.10	0.91	19.45±1.92	35.99±4.20	0.88	56.29±1.77	0.23±0.02	0.94	100	0.06±0.01	0.49±0.03	0.78±0.04	0.98

Zero-order: $y=ax+b$

First-order: $y=a*(1-e^{-b})$

Higuchi: $y=a*\sqrt{x}+b$

Korsmeyer-Peppas: $y=a*x^{b}$

Weibull models: $y=A(1-e^{-(k(x-x_c))^{d}})$
